# The safety and feasibility of transoral thyroidectomy vestibular approach in the treatment of thyroid disorders: An overview of systematic reviews

**DOI:** 10.1371/journal.pone.0326318

**Published:** 2025-07-02

**Authors:** Huixia Ren, Mengyang Wang, Xuan Sun, Yin Yuan, Naijin Zhang, Ying Liu, Shujiao Yue, Yonghui Li, Yuanyuan Guan, Huaien Bu, Hongwu Wang

**Affiliations:** 1 School of Public Health, Tianjin University of Traditional Chinese Medicine, Tianjin, China; University Putra Malaysia, MALAYSIA

## Abstract

**Objective:**

Many systematic reviews (SRs) and meta-analyses (MAs) have recently assessed the short-term outcomes of the transoral thyroidectomy vestibular approach (TOTVA) compared with conventional open thyroidectomy (COT) and non-transoral endoscopic thyroidectomy (NTET). However, their conclusions remain controversial. This overview aimed to evaluate the safety and feasibility of TOTVA by appraising the quality of existing SRs/MAs.

**Methods:**

Seven Chinese and English databases were systematically searched from their inception to December 10, 2023. Eligible SRs/MAs, published between 2020 and 2023, compared the safety and efficacy of TOTVA with COT or NTET. The PRISMA, AMSTAR-2, and ROBIS tools were used to assess reporting quality, methodological quality, and risk of bias, respectively.

**Result:**

Eleven SRs/MAs were finally included. According AMSTAR-2, one study was assessed as high-quality, with the remainder as very low-quality. Using PRISMA 2020, the “Yes” response rate for Q5, Q8, and Q15 was below 55 percent. Per ROBIS, all SRs/MAs exhibited low risk in phase 1 and domain 1 but high risk in domain 2. Efficacy was assessed through intraoperative outcomes, primary postoperative outcomes, and statistically significant postoperative outcomes. Patients with thyroid disorders undergoing TOTVA experienced longer overall operative time and hospital stays, reduced intraoperative blood loss, increased lymph node retrieval, higher incidence of infection, lower postoperative pain scores, reduced incidence of hypocalcemia, larger drainage volumes, and higher cosmetic effect scores compared with those undergoing.

**Conclusion:**

The TOTVA may enhance cosmetic satisfaction, improve lymph node retrieval, and decreased postoperative complications. Nevertheless, these findings warrant cautious interpretation due to low methodological quality, high risk of bias, and limited evidence quality. More rigorous and standardized SRs/MAs are required to provide robust scientific evidence for definitive conclusions.

## 1. Introduction

Advancements in global health awareness and diagnostic techniques, such as ultrasound and fine-needle aspiration biopsy, have led to an increased detection rate of thyroid nodules in recent years [1]. These nodules are broadly classified as benign or malignant [2,3]. Surgical intervention remains the most effective treatment for thyroid carcinoma, with conventional open thyroidectomy (COT) long established as the standard approach for patients undergoing thyroidectomy [4,5]. However, COT results in a postoperative cervical scar of at least 4 cm, often accompanied by complications such as hyperpigmentation, which may be undesirable for patients prioritizing cosmetic outcomes [6,7]. To address these cosmetic and procedural concerns, minimally invasive techniques have been developed, including transoral [8], axillo-breast [9], axillary [10], anterior chest [11], areola [12], and retroauricular approaches [13]. Although these techniques, except for the transoral approach, are less invasive than COT, most still require visible external incisions that may result in noticeable scarring [14]. In addition, such approaches may necessitate extensive flap dissection [15], limit access to central neck lymph node dissection [16], or pose challenges in preserving the parathyroid glands during surgery [17].

The transoral thyroidectomy vestibular approach (TOTVA), first documented in 2016 [18], encompasses the transoral endoscopic thyroidectomy vestibular approach (TOETVA) and the transoral robotic thyroidectomy vestibular approach (TORTVA). Recognized as the only truly scarless technique, TOTVA ensures surgical quality while effectively treating thyroid disorders [19,20]. Compared with COT and non-transoral endoscopic thyroidectomy (NTET), TOTVA offers advantages such as reduced tissue dissection and no cutaneous scarring, making it an appealing treatment option for patients with specific thyroid disorders [21,22].

Thyroidectomy has been extensively studied in evidence-based medical research [23]. Numerous systematic reviews (SRs) and meta-analyses (MAs) have compared outcomes of TOTVA with those of COT and NTET, yet findings remain inconsistent or contradictory. For instance, a meta-analysis by Wang *et al*. [24] reported larger postoperative drainage volumes in patients undergoing TOETVA compared with those undergoing COT, whereas Zhou’s analysis [25] showed that the TOETVA group gained less drainage volume than the anterior chest approach (ACA) group. Similarly, Xia *et al*. [12] noted significantly less intraoperative blood loss in the TOETVA group compared with the endoscopic thyroidectomy via the areolar approach (ETAA) group, while Zhou *et al*. [26] reported increased blood loss in the TOTVA group compared with the COT group. These discrepancies render the comparison of outcomes between TOTVA and COT or NTET clinically contentious.

SRs/MAs provide an objective synthesis of published literature on a specific topic through rigorous and systematic methodology [27,28]. Positioned at the apex of the evidence pyramid, high-quality SRs/MAs offer reliable guidance for evidence-based medicine. However, low-quality SRs/MAs may mislead clinical decision-making [29]. To date, no overview of SRs/MAs has evaluated the outcomes of TOTVA compared with COT and NTET in the treatment of thyroid disorders. This review comprehensively analyzed relevant SRs/MAs to critically assess the safety and feasibility of TOTVA compared with COT and NTET. The objectives were to (1) summarize the efficacy and safety outcomes reported in SRs/MAs comparing TOTVA with COT and NTET, (2) evaluate the methodological quality of the included SRs/MAs, and (3) identify inconsistencies or uncertainties in the evidence to provide a reference for future research and clinical practice.

## 2. Methods and methods

### 2.1 Registration

Due to the study design, ethical approval was not required. The study protocol was registered in the International Prospective Register of Systematic Reviews (PROSPERO) [30] under the registration number CRD42023475698.

### 2.2 Inclusion and exclusion criteria

#### 2.2.1. Study type.

SRs/MAs evaluating the TOTVA compared with COT or NTET for the treatment of thyroid disorders were included, regardless of study geography. Eligible SRs/MAs, published in Chinese and English, incorporated randomized controlled trials (RCTs) and non-randomized studies.

#### 2.2.2. Subjects.

Patients requiring thyroidectomy were included, irrespective of geography, race, disease duration, or other factors.

#### 2.2.3. Interventions.

The experimental group underwent TOTVA, while the control group underwent COT or NTET, encompassing conventional open, axillo-breast, axillary, anterior chest, areolar, retroauricular, or other approaches.

#### 2.2.4. Outcome indicators.

Primary intraoperative outcomes included operative time, whereas secondary outcomes comprised the number of retrieved central lymph nodes (CLNs), metastatic lymph nodes (MLNs), and blood loss volume. The primary postoperative outcome was hospital stay (days), with secondary outcomes including at least one of the following: postoperative pain, recurrent laryngeal nerve (RLN) injury, hypocalcemia, seroma, wound infection, drainage, or hematoma.

#### 2.2.5. Exclusion criteria.

Studies were excluded if they met the following criteria: (i) duplicate publications; (ii) conference papers, abstracts, expert consensus, case reports, or reviews; (iii) inclusion of patients with a history of neck surgery; (iv) absence of primary reported outcomes; or (v) incorrect or non-extractable data.

### 2.3. Retrieval strategy

Two reviewers (RHX and ZNJ) systematically searched PubMed, EMBASE, Web of Science, Cochrane Library, China Knowledge Resource Integrated Database (CNIK), VIP Database, and WANFANG Data for SRs/MAs comparing TOETVA with COT or NTET in the treatment of thyroid-related diseases, from database inception to December 10, 2023. Search terms included synonyms and combinations of “thyroidectomy.” The detailed search strategy for each database is presented in S1 Appendix A.

### 2.4. Literature screening and data extraction

Retrieved SRs/MAs were imported into Endnote X9, and duplicates were removed. Two reviewers (XS and YY) independently screened titles and abstracts to identify potentially relevant studies and determined eligibility based on full-text reviews. Discrepancies were resolved through discussion with a third reviewer. The search process was conducted according to the PRISMA 2020 flow diagram (S2 Appendix B) [31]. Concurrently, two reviewers (LY and YSJ) independently extracted data, including first author, publication year, country, literature size, sample size, interventions, control groups, methodological assessment tools, outcome indicators, and funding sources.

### 2.5. Assessment methods

#### 2.5.1. Reporting quality evaluation tool—PRISMA (S3 Appendix C).

The reporting quality of included SRs/MAs was evaluated using PRISMA 2020 [32,33], which comprises 27 items across seven domains. Each item was scored as “Yes” (1 point), “Partially Yes” (0.5 points), or “No” (0 points). Reports were classified as relatively complete (score 21–27), slightly incomplete (score 15–21), or seriously deficient (score ≤ 15). Two reviewers (LYH and ZNJ) independently assessed reporting quality, with a third reviewer (WMY) resolving any discrepancies.

#### 2.5.2. Methodological quality assessment tool—AMSTAR-2 scale (S4 Appendix D).

The methodological quality of SRs/MAs was assessed using AMSTAR-2 [34,35], which includes 16 items rated as “Yes,” “Partially Yes,” or “No.” Studies were categorized as high, moderate, low, or very low quality. The following are key items: 2, 4, 7, 9, 11, 13, and 15. Two reviewers (LYY and ZNJ) independently evaluated methodological quality, and disagreements were resolved by a third reviewer (WMY).

#### 2.5.3. Risk of bias assessment tool — ROBIS scale (S5 Appendix E).

The ROBIS scale [36,37] was used to assess the risk of bias in SRs/MAs across three phases: (1) relevance assessment, (2) identification of review process concerns, and (3) overall risk of bias judgment. Results were categorized as “low,” “unclear,” or “high.” Two reviewers (LYY and ZNJ) independently evaluated the risk of bias in the SRs/MAs, with discrepancies resolved by a third reviewer (WMY).

### 2.6. Data synthesis

Characteristics of included SRs/MAs, along with results from AMSTAR-2, PRISMA, and ROBIS assessments, are summarized in the charts and tables.

## 3. Results

### 3.1. Literature retrieval results

**Fig 1:**
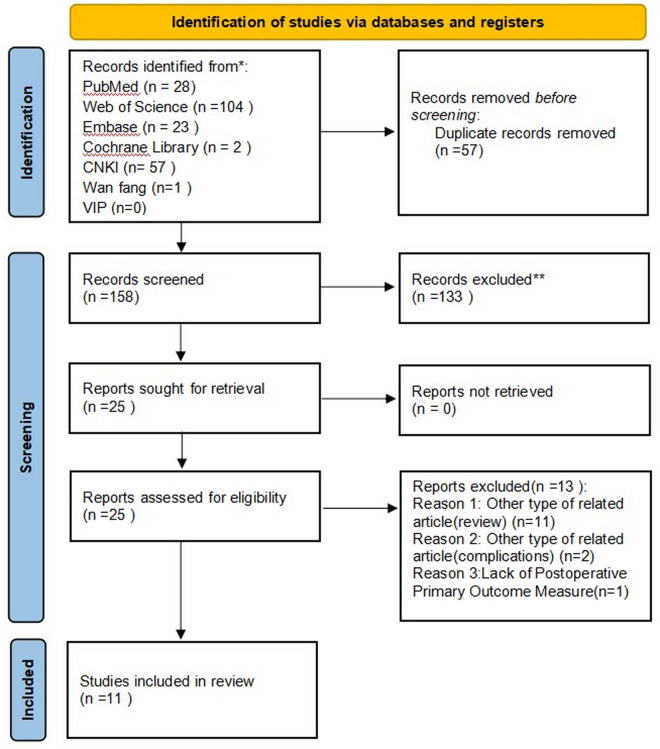
Flowchart of the screening process.

A total of 215 studies were retrieved using the search strategy, including 28 from PubMed, two from Cochrane, 23 from Embase, 104 from WOS, 55 from CNKI, one from Wanfang, and none from VIP. After excluding 57 duplicate publications, 158 studies remained. Following screening of titles, abstracts, and full texts, 11 SRs/MAs [12,14,24–27,38–42] were included in this overview. The study selection process is illustrated in Fig 1, with details of included SRs/MAs and exclusion criteria provided in S6 Appendix F.

### 3.2. Basic features of the included literature

Of the 11 included SRs/MAs, 1 was a dissertation [41], while the others [12,14,24–27,38–40,42] were published in journals. Eight [12,14,24,26,27,38–40] were published in English, and three [25,41,42] in Chinese, spanning 2020–2023. Authors were from Kuwait (n = 1), the USA (n = 1), Korea (n = 1), and China (n = 9). Each SR or Ma included 5–15 experiments, with sample sizes ranging from 689 to 3048 per trial. All experimental interventions involved the TOTVA, while control interventions included COT or NTET, such as conventional open, axillo-breast, axillary, anterior chest, areolar, and retroauricular approaches. Regarding risk of bias assessment tools, one [38] used the Bias in Non-Randomized Studies (ROBINS-I) tool, one [39] used the Cochrane Collaboration’s tool and the Newcastle-Ottawa Scale, one [40] used the GRADE Pro Software, and the remaining eight [12,14,24–26,40–42] used the Newcastle-Ottawa Scale. Detailed study characteristics are presented in Table 1.

**Table 1:** Characteristics of included reviews.

First author/Year	Country	Study size/number of cases	Design(R/P/RCT)	Pathology	Therapy group	Control group	Methodology evaluation tools	Outcomes	Funding
Xia et al. 2022 [12]	China	10/934	7/0/3	a(3)/b(5)/f(1)	TOETVA	ETAA	Newcastle-Ottawa Scale	①②⑤⑦⑯⑰㉘㉙㉚㉛	(7)(8)
Huo et al. 2023 [14]	China	12/3048	10/0/2	a(7)/abg(1)/bg(1)g(1)	TOETVA	COT	Newcastle Ottawa Scale	①②③⑤⑥⑧⑩⑪⑬⑭⑮⑯㉗	(2)(3)(4)(5)
Wang et al. 2021 [24]	China	6/1151	5/1/0	a(3)/bg(3)	TOETVA	COT	Newcastle-Ottawa Scale	①②③④⑤⑥⑩⑪⑬⑭⑮⑯	(9)
Zhou et al. 2022 [25]	China	7/689	7/0/0	c(7)	TOETVA	Transcervical thyroidectomy	Newcastle-Ottawa Scale	①②③⑤⑥⑨⑯㉓㉖㉜	Unknown
Zhou et al. 2023 [26]	China	15/2955	13/2/0	a(6)/ab(1)/bg(6)/bcd(1)/f(1)	TOETVA	COT	Newcastle-Ottawa Scale	①②③④⑤⑥⑩⑪⑮⑯㉔㉕	(6)
Oh et al. 2023 [27]	Korea	13/2889	13/0/0	a(4)/b(2)gb(6)/F(1)	TOETVA	COT	GRADE Pro Software	①②③④⑤⑥⑦⑩⑪⑬⑭⑮	None
Albazee et al. 2023 [38]	Kuwait	5/923	3/2/0	a(1)/ab(1)/abd(1)/bc(2)	TORTVA	Bilateral axillo-breast approach-robotic thyroidectomy (BABA-RT)	Risk of Bias in Non-Randomized Studies (ROBINS-I)	①③⑤⑦⑩⑬⑭	None
Dabsha et al. 2022 [39]	USA	15/2173	11/3/1	a(5)/b(4)/ab(1)/ac(1)/bc(1)/abd(1)/e(1)/bf(1)	transoral robotic thyroidectomy and TOETVA	NTET(Trans-axillary, Trans-areolar, or Combined axillo-areolar approach)	Cochrane collaboration’s tool and Newcastle-Ottawa Scale	①②③⑤⑥⑦⑨⑫⑱⑲⑳㉑	None
Wang et al. 2021 [40]	China	10/1677	10/0/0	a(4)/b(1)/ab(1)//abc(1)//abd(1)/bfg(1)/fg(1)	TOETVA	NTET, including subclavian, areola, trans-axillary, breast, axillo-breast and dorsal approach	Newcastle-Ottawa Scale	①②③⑤⑥⑧⑩⑪⑬㉒	(1)
Wei 2022 [41]	China	8/1352	8/0/0	a(6)/b(1)/c(1)	TOETVA	COT	Newcastle-Ottawa Scale	①②③⑤⑥⑩⑬㉝	Unknown
Xue et al. 2020 [42]	China	7/1465	7/0/0	a(7)	TOETVA	COT	Newcastle-Ottawa Scale	①②③⑤⑩⑪⑬⑭⑮	(10)

Note: Design: ROS: retrospective; PRO: prospective; RAN: random; Pathology: a: PTC, papillary thyroid cancer; b: BTN, benign thyroid nodule; c: DTC, diferentiated thyroid cancer; d: FT, follicular tumor; e: TMC, thyroid microcarcinoma; f: Graves’ disease; g: Malignant tumors. Outcome measures: ①operative time; ②blood loss; ③number of retrieved central lymph nodes; ④number of metastatic lymph node; ⑤hospital stay; ⑥wound infection; ⑦postoperative pain; ⑧seroma; ⑨recurrent laryngeal nerve injury; ⑩Transient recurrent laryngeal nerve injury; ⑪Permanent recurrent laryngeal nerve injury; ⑫hypocalcemia; ⑬Transient hypocalcemia; ⑭permanent hypocalcemia; ⑮hematoma; ⑯drainage volume; ⑰drainage time(d); ⑱temporary hoarseness; ⑲Any hoarseness (temporary or permanent); ⑳cost; ㉑chyle leak; ㉒Unintended parathyroidectomy; ㉓Parathyroid gland injury; ㉔Transient hypoparathyroidism; ㉕permanent hypoparathyroidism; ㉖superior laryngeal nerve injury; ㉗chylous fifistula; ㉘Postoperative WBC; ㉙Postoperative CPR; ㉚cosmetic effect score; ㉛satisfaction score; ㉜CO_2_ embolism; ㉝recurrence within 1 year; Funding: (1)Sichuan Science and Technology Program (No. 2019YJ0038); (2) The Department of Science and Technology ofGuizhou Province through Grant No. Qiankehe Foundation—ZK(2023) General 485; (3)The National Natural Science Foundation of China (NSFC) through Grant No. 81960125; (4)The Department of Science and Technology of Guizhou Province through Grant No. Qiankehe Foundation (2020) 1Y302; (5)The Department of Science and Technology Department of Guizhou Province through Grant No. LC (2021) 004; (6)Sichuan Science and Technology Program (No. 2019YJ0038); (7)Project for Disciplines of Excellence (ZY2017309), West China Hospital, Sichuan University; (8)Project of Science and Technology Department of Sichuan Province(2021YFS0103), West China Hospital, Sichuan University; (9)Sichuan Science and Technology Program (No. 2019YJ0038); (10)National Natural Science Foundation of China (81572916).

### 3.3. Reporting the quality evaluation results

#### 3.3.1. Report quality.

The structure of included studies in the sections of Title, Abstract, Introduction, Result, Discussion, and Funding were relatively complete, with a response rate of over 80% for “Yes” or “Partially Yes” across 21 projects. For the remaining six items, three had a “Yes” or “Partially Yes” response rate below 55%; only four SRs/MAs [27,38,39,42] reported prior study registration (item 5); six SRs/MAs [12,27,38,39,41,42] provided a comprehensive search strategy for at least one electronic database; and six SRs/MAs [12,14,26,27,40,41] addressed potential bias in composite results (item 15). Additional details are provided in Table 2.

**Table 2:** Result of the PRISMA assessments.

Section/topic	Items	Xia et al. 2022 [12]	Huo et al. 2023 [14]	Wang et al. 2021 [24]	Zhou et al. 2022 [25]	Zhou et al. 2023 [26]	Oh et al. 2023 [27]	Albazee et al. 2023 [38]	Dabsha et al. 2022 [39]	Wang et al. 2021 [40]	Wei 2022 [41]	Xue et al. 2020 [42]	Compliance (%)
Title	Q1. Title	Y	Y	Y	Y	Y	Y	Y	Y	Y	Y	Y	100
Abstract	Q2. Structured summary	Y	Y	Y	Y	Y	Y	Y	Y	Y	Y	Y	100
Introduction	Q3. Rationale	Y	Y	Y	Y	Y	Y	Y	Y	Y	Y	Y	100
Methods	Q4. Objectives	Y	Y	Y	Y	Y	Y	Y	Y	Y	Y	Y	100
	Q5. Protocol and registration	N	N	N	N	N	Y	Y	Y	N	N	Y	36.36
	Q6. Eligibility criteria	Y	Y	Y	Y	Y	Y	Y	Y	Y	Y	Y	100
	Q7. Information sources	Y	Y	Y	Y	Y	Y	Y	Y	Y	Y	Y	100
	Q8. Search	Y	N	N	N	N	Y	Y	Y	N	Y	Y	54.54
	Q9. Study selection	Y	Y	Y	Y	Y	Y	Y	Y	Y	Y	Y	100
	Q10. Data collection process	Y	PY	Y	PY	Y	Y	Y	Y	Y	Y	Y	81.81
	Q11. Data items	Y	Y	Y	PY	Y	Y	Y	Y	Y	PY	Y	100
	Q12. Risk of bias in individual studies	Y	Y	Y	Y	Y	Y	Y	Y	Y	Y	Y	100
	Q13. Summary measures	Y	Y	Y	Y	Y	Y	Y	Y	Y	Y	Y	100
	Q14. Synthesis of results	Y	Y	Y	Y	Y	Y	Y	Y	Y	Y	Y	100
	Q15. Risk of bias across studies	Y	Y	N	N	Y	Y	N	N	Y	Y	N	54.54
	Q16. Additional analyses	Y	Y	Y	Y	Y	Y	Y	N	Y	N	Y	81.81
Methods	Q17. Study selection	Y	Y	Y	Y	Y	Y	Y	N	Y	Y	Y	90.90
	Q18. Study characteristics	Y	Y	Y	Y	Y	Y	Y	Y	Y	Y	Y	100
	Q19. Risk of bias within studies	Y	Y	Y	Y	Y	Y	Y	Y	Y	Y	Y	100
	Q20. Results of individual studies	Y	Y	Y	Y	Y	Y	Y	PY	Y	Y	Y	100
	Q21. Synthesis of results	Y	Y	Y	Y	Y	Y	Y	Y	Y	Y	Y	100
	Q22. Risk of bias across studies	Y	Y	Y	N	Y	Y	N	N	Y	Y	N	63.63
	Q23. Additional analysis	Y	Y	Y	N	Y	Y	N	N	Y	Y	Y	72.72
Discussion	Q24. Summary of evidence	PY	PY	PY	PY	PY	PY	PY	PY	PY	PY	PY	100
	Q25. Limitations	Y	Y	Y	Y	Y	Y	Y	Y	Y	Y	Y	100
	Q26. Conclusions	Y	Y	Y	Y	Y	N	Y	N	Y	N	Y	72.72
	Q27. Funding	Y	Y	Y	N	Y	Y	Y	Y	Y	N	Y	81.81

Note: Y: Yes; PY: partial Yes; N: No

#### 3.3.2. Methodological quality.

Using AMSTAR-2, one of the included studies [27] was rated as high quality, while the remaining 10 SRs/MAs [12,14,24–26,38–42] were rated as very low quality.

Among critical items, four SRs/MAs [27,38,39,42] reported prior registration (item 2); none searched relevant grey literature (item 4); only one [27] provided a complete list of excluded studies with justifications (item 7); three [14,26,39] did not account for potential bias in study inclusion (item 13); and five [12,14,24,26,38–42] failed to consider publication bias (item 15). Non-critical items were generally well-addressed. Complete details are presented in Table 3.

**Table 3:** Result of the AMSTAR-2 assessments.

Author/year	Q1	Q2*	Q3	Q4*	Q5	Q6	Q7*	Q8	Q9*	Q10	Q11*	Q12	Q13*	Q14	Q15*	Q16	Quality
Xia et al. 2022 [12]	Y	N	Y	PY	Y	Y	N	Y	Y	N	Y	Y	Y	Y	Y	Y	CL
Huo et al. 2023 [14]	Y	N	Y	PY	Y	Y	N	Y	Y	N	Y	Y	N	Y	Y	Y	CL
Wang et al. 2021 [24]	Y	N	Y	PY	Y	Y	N	Y	Y	N	Y	Y	Y	Y	Y	Y	CL
Zhou et al. 2022 [25]	Y	N	Y	PY	Y	Y	N	Y	Y	N	Y	Y	Y	Y	N	N	CL
Zhou et al. 2023 [26]	Y	N	Y	PY	Y	Y	N	Y	Y	N	Y	Y	N	Y	Y	Y	CL
Oh et al. 2023 [27]	Y	Y	Y	PY	Y	Y	Y	Y	Y	Y	Y	Y	Y	Y	Y	Y	H
Albazee et al. 2023 [38]	Y	Y	Y	PY	Y	Y	N	Y	Y	N	Y	Y	Y	Y	N	Y	CL
Dabsha et al. 2022 [39]	Y	Y	Y	PY	Y	Y	N	Y	Y	N	Y	Y	N	Y	N	Y	CL
Wang et al. 2021 [40]	Y	N	Y	PY	Y	Y	N	Y	Y	N	Y	Y	Y	Y	Y	Y	CL
Wei 2022 [41]	Y	N	Y	PY	Y	Y	N	Y	Y	N	Y	Y	Y	Y	Y	N	CL
Xue et al. 2020 [42]	Y	Y	Y	PY	Y	Y	N	Y	Y	N	Y	Y	N	N	N	Y	CL

Note: Y: Yes; PY: partial Yes; N: No

#### 3.3.3. Risk of bias.

Per the ROBIS scale, all SRs/MAs were rated as having low risk of bias in phase 1 and domain 1 of Phase 2. However, all SRs/MAs exhibited high risk of bias in domain 2, 4 in Domain 3, and 5 in Domain 4. In Phase 3, 7 SRs/MAs were classified as low risk, whereas the remaining four were rated as high risk. Additional details are provided in Table 4.

**Table 4:** Result of the ROBIS assessments.

Review	Phase 1	Phase 2				Phase 3
	Assessing relevance	Domain 1. Study eligibility criteria	Domain 2. Identification and selection of studies	Domain 3. Data collection and study appraisal	Domain 4. Synthesis and findings	Risk of bias in the review
Xia et al. 2022 [12]	L	L	H	L	L	L
Huo et al. 2023 [14]	L	L	H	L	L	L
Wang et al. 2021 [24]	L	L	H	L	L	L
Zhou et al. 2022 [25]	L	L	H	H	H	H
Zhou et al. 2023 [26]	L	L	H	L	L	L
Oh et al. 2023 [27]	L	L	H	L	L	L
Albazee et al. 2023 [38]	L	L	H	H	H	H
Dabsha et al. 2022 [39]	L	L	H	H	H	H
Wang et al. 2021 [40]	L	L	H	L	L	L
Wei 2022 [41]	L	L	H	L	H	L
Xue et al. 2020 [42]	L	L	H	H	H	H

Note: L: Low; H: High

#### 3.3.4. Efficacy evaluation results.

Intraoperative, primary postoperative, and statistically significant postoperative outcomes were summarized. Operative time was reported in all SRs/MAs, with eight indicating longer operative times for patients undergoing TOTVA compared with those undergoing COT or NTET. Blood loss and the number of retrieved CLNs were reported in 10 SRs/MAs, with five demonstrating reduced intraoperative blood loss, and four showing increased CLN retrieval in patients undergoing TOTVA compared with those undergoing COT or NTET (P < 0.05). MLNs were reported in three SRs/MAs, with no significant differences between groups (P > 0.05). Hospital stay was reported in all SRs/MAs, with five indicating longer hospital stays for patients undergoing TOTVA compared with those undergoing COT/NTET group (P < 0.05). Wound infection was reported in one SR/MA, showing a significant increase in patients undergoing TOTVA (P < 0.05). Regarding postoperative pain, two SRs/MAs were statistically significant, both reporting significantly lower pain scores in patients undergoing TOTVA compared with those undergoing COT or NTET (P < 0.05). Only one SR/MA reported a substantial difference that favored the TOTVA group over the COT/NTET group (P < 0.05). Four SRs/MAS revealed a higher drainage volume in the TOTVA group (P < 0.05). Finally, one SR/Ma reported a greater satisfaction score in patients undergoing TOTVA (P < 0.05). Additional details regarding the efficacy assessment is presented in Table 5.

**Table 5:** A. Quality of evidence: TOTVA vs. COT. B. Quality of evidence: TOTVA vs. NTET (including robotic approaches).

Study ID	Comparison Type	Outcomes	Pooled effect size(95% CI)	I^2^%	P value
Huo et al. 2023 [14]	TOTVA vs COT	operative time	MD = 73.64(49.34,97.94)	99	<0.00001
		blood loss	MD = 0.92(−1.40,3.23)	0	0.44
		number of retrieved central lymph nodes	MD = −1.42(−2.82,-0.03)	90	0.05
		hospital stay	MD = 0.28(0.18,0.38)	1	<0.00001
		drainage volume	MD = 91.0(35.52,146.48)	96	0.001
Wang et al. 2021 [24]	TOTVA vs COT	operative time	MD = 66.09(35.22,96.96)	98	<0.0001
		blood loss	MD = 0.93(−1.54,3.39)	32	0.46
		number of retrieved central lymph nodes	MD = 0.29(−1.35,1.93)	73	0.73
		number of metastatic lymph nodes	MD = 0.32(−0.00,0.65)	0	0.05
		hospital stay	WMD = 0.02(−0.50,0.54)	NA	0.94
		drainage volume	MD = 98.00(20.14,175.86)	99	0.01
Zhou et al. 2023 [26]	TOTVA vs COT	operative time	MD = 66.86(47.15,86.56)	99	<0.00001
		blood loss	MD = 2.83(1.77,3.90)	49	<0.00001
		number of retrieved central lymph nodes	WMD = −0.74(−1.94,0.46)	80	0.23
		number of metastatic lymph nodes	MD = 0.16(−0.10,0.43)	45	0.23
		hospital stay	MD = 0.20(0.01,0.39)	70	0.04
		wound infection	OR=5.62(1.57,20.10)	28	0.008
		drainage volume	MD = 79.42(37.88,120.96)	96	<0.00001
Oh et al. 2023 [27]	TOTVA vs COT	operative time	MD = 55.19(39.15,71.23)	97	<0.00001
		blood loss	MD = 1.76(−0.83,4.35)	75	0.18
		number of retrieved central lymph nodes	MD = −061(−1.84,0.63)	79	0.33
		number of metastatic lymph nodes	SMD = −0.08(−0.39,0.24)	82	0.63
		hospital stay	MD = 0.27(0.14,0.39)	43	<0.0001
		postoperative pain	MD = −1.41(−2.79,-0.03)	99	0.04
Wei 2022 [41]	TOTVA vs COT	operative time	MD = 60.78(58.09,63.47)	99	<0.00001
		blood loss	MD = 3.63(3.06,4.20)	85	<0.00001
		number of retrieved central lymph nodes	MD = 1.34(1.11,1.57)	68	<0.00001
		hospital stay	MD = −0.65(−0.75,-0.56)	99	<0.00001
		Transient hypocalcemia	OR=0.31(0.17,0.58)	47	0.0002
Xue et al. 2020 [42]	TOTVA vs COT	operative time	MD = 35.18(17.45,52.90)	99	0.0001
		blood loss	MD = −5.32(−17.41,6.78)	97	0.39
		number of retrieved central lymph nodes	MD = 1.42(0.62,2.21)	62	0.0005
		hospital stay	MD = 0.05(−1.31,1.41)	98	0.94
**B. Quality of evidence: TOTVA vs. NTET (including robotic approaches)**					
Study ID	Comparison Type	Outcomes	Pooled effect size(95% CI)	I^2^%	P value
Xia et al. 2022 [12]	TOETVA vs ETAA	operative time	MD = 4.92(−4.74,14.58)	94	0.32
		blood loss	MD = −1.32(−2.44,-0.21)	10	0.02
		hospital stay	MD = 0.02(−0.16,0.20)	74	0.83
		Cosmetic effect score	MD = 1.22(0.43,2.00)	97	0.002
Zhou et al. 2022 [25]	TOETVA vs Transcervical thyroidectomy	operative time	MD = −0.95(−2.79,0.90)	0	0.31
		blood loss	MD = −1.62(−2.70,-0.54)	0	0.003
		number of retrieved central lymph nodes	MD = 1.08(0.07,2.08)	93	0.04
		hospital stay	MD = 0.41(−0.59,1.41)	96	0.42
		drainage volume	MD = −9.85(−17.82,-1.88)	95	0.02
Albazee et al. 2023 [38]	TORTVA vs BABA-RT	operative time	MD = 36.27(−6.87,79.41)	97	>0.10
		number of retrieved central lymph nodes	MD = 0.42(−0.16,0.99)	0	0.16
		hospital stay	MD = −0.14(−0.66,0.38)	93	0.60
		Transient hypocalcemia	MD = 0.07(0.02,0.25)	0	<0.0001
		postoperative pain			
		postoperative pain Day 0	MD = −0.64(−0.95,-0.33)	88	<0.0001
		postoperative pain Day 1	MD = −0.43(−0.60,-0.26)	84	<0.0001
		postoperative pain Day 2	MD = −0.30(−0.55,-0.06)	85	0.02
		postoperative pain Day 3	MD = 0.03(−0.51,0.58)	88	0.9
Dabsha et al. 2022 [39]	transoral robotic thyroidectomy and TOETVA vs NTET	operative time	SMD = 0.72(0.07,1.37)	98	<0.01
		blood loss	SMD = −0.26(−0.43,-0.09)	0	0.001
		number of retrieved central lymph nodes	SMD = 0.32 (0.02,0.62)	84.9	0.03
		hospital stay	SMD = −0.27(−0.83,0.29)	96.6	0.34
Wang et al. 2021 [40]	TOETVA vs NTET	operative time	MD = 22.6(7.51,37.69)	98	0.003
		blood loss	MD = 0.48(−1.19,2.16)	46	0.57
		number of retrieved central lymph nodes	MD = 0.49(−1.12,2.11)	86	0.55
		hospital stay	OR=0.80(0.42,1.50)	51	0.49

Note: NA: not available, OR: odd ratios,95% CI: confidence intervals, WMD: weighted mean difference, SMD: standard mean difference, MD: mean difference, RD: risk difference.

## 4. Discussion

### 4.1. Main findings

This overview analyzed 11 SRs/MAs published between 2020 and 2023 to compare the safety and efficacy of the TOTVA with COT and NTET. The recent publication of these studies reflects growing interest in this research area. The included SRs/MAs suggest that TOTVA is a safe and feasible surgical option, comparable to COT and NTET, for patients with certain thyroid disorders, including benign thyroid nodules, selected papillary thyroid cancer, and differentiated malignant thyroid cancer. Differences were observed in intraoperative outcomes, such as operative times, blood loss, lymph node retrieval, as well as postoperative complications. However, the methodological and reporting quality of these SRs/MAs requires rigorous evaluation to ensure reliable conclusions. This overview aims to guide the development of high-quality SRs/MAs by identifying deficiencies.

Cosmetic satisfaction was reported to be higher in patients undergoing TOTVA compared with those undergoing COT or NTET in one SR/MA [12]. Additionally, a trend toward increased retrieval of CLNs and MLNs intraoperatively, along with a lower incidence of postoperative hypocalcemia, was noted in patients undergoing TOTVA compared with those undergoing COT or NTET in several SRs/MAs. However, according to the AMSTAR-2 assessment, only one SR/MA was rated as high quality, with the remaining 10 [12,14,24–26,38–42] classified as very low quality, particularly in items 2, 4, and 10, which can considerably impair the authenticity of SRs/MAs and the reliability of the evidence. Similar deficiencies were identified in the PRISMA assessment, including lack of prior registration, incomplete search strategies, absence of excluded study lists, and inadequate analysis of publication bias. These issues contributed to suboptimal reporting quality. The ROBIS assessment highlighted biases primarily in the retrieval and screening processes, such as the omission of grey literature, and in the lack of sensitivity analyses or explanations for heterogeneity in some studies. Consequently, SRs/MAs with higher methodological quality and lower risk of bias are required to verify the safety and feasibility of TOTVA for patients with thyroid disorders. Future studies should prioritize randomization, allocation concealment, blinding, and larger sample sizes.

### 4.2. Strengths and limitations

This overview is the first to synthesize SRs/MAs comparing intraoperative and postoperative outcomes of TOTVA with COT and NTET. Seven Chinese and English databases were searched, and the AMSTAR-2, PRISMA 2020, and ROBIS scales were used to comprehensively evaluate the quality of included SRs/MAs. Nevertheless, several limitations must be acknowledged. First, the complexity of robotic surgery systems may prolong operative times, and variations in surgeons’ skills could introduce selective bias when pooling effect sizes. Second, the subjective nature of PRISMA 2020, AMSTAR-2, and ROBIS assessments may have introduced bias in the evaluation process. Third, the inclusion of only Chinese and English literature, excluding databases languages such as German, Korean, or Japanese, may have resulted in language bias. Fourth, among the included SRs/MAs, nine [12,14,24–26,39,41–43] were assessed using the Newcastle-Ottawa Scale (NOS), which is designed for case-control and cohort studies. However, most studies within these SRs/MAs were retrospective, and only three [12,14,39] included a limited number of RCTs. The use of ROBINS-I and ROS scales would have been more appropriate, potentially affecting result reliability. Finally, some of the studies included in the nine SRs/MAs [12,14,24,26,27,38–41] did not distinguish between malignant and benign thyroid tumors, and four [12,27,38,39] lacked specific inclusion criteria regarding tumor type, which may confound interpretation of the findings.

### 4.3. Implications for future research

The central-median approach of TOTVA, with its top-down surgical view [43], facilitates exposure of the thyroid gland, parathyroid glands, and surrounding tissues without obstruction from the sternum or clavicle. This approach may enhance lymph node retrieval and reduce tissue separation and surgical trauma [44]. Furthermore, the fourth-generation da Vinci® SP robotic system can effectively filter hand tremors, magnify vision, and allow precise movements and identification through the robotic arms equipped with flexible wrists and fingers during cervical lymph node dissection. In addition, this method requires only a small incision and minimal working space. These findings suggest that TOTVA is a valuable alternative to COT and NTET, offering favorable clinical and cosmetic outcomes.

Several avenues for future research are proposed. First, to enhance methodological and reporting quality, SRs/MAs should be registered in advance to ensure transparency and avoid duplication. Second, inclusion criteria should be refined to separately evaluate TOETVA and TORTVA, enabling more precise assessments of their characteristics, efficacy, and safety. Third, clear distinctions between malignant and benign thyroid tumors should be incorporated into inclusion criteria to mitigate confounding effects. Fourth, larger sample sizes in the original studies, particularly high-quality RCTs, are needed to minimize bias and enhance generalizability. Fifth, expanding literature searches to include languages beyond Chinese and English would reduce language bias. Sixth, appropriate risk-of-bias tools should be used to ensure systematic and standardized evaluations. Seventh, researchers should provide a list of excluded studies with justifications to increase transparency and reliability. Finally, to address significant heterogeneity, subgroup or meta-regression analyses should be conducted to strengthen the credibility of the findings.

## 5. Conclusions

The safety profile of TOTVA is comparable to that of COT and NTET. Potential benefits include reduced postoperative pain, increased retrieval of CLNs and MLNs, lower rates of transient hypocalcemia, and improved cosmetic outcomes in selected patients. Nevertheless, the low methodological quality, limited evidence quality, high risk of bias, and significant heterogeneity in the included SRs/MAs necessitate cautious interpretation of these findings. Future large-scale, high-quality, multicenter studies are required to provide robust and standardized evidence supporting the feasibility and efficacy of TOTVA.

## Supporting information

S1 Appendix A Search strategy. (DOCX)

S2 Appendix B PRISMA 2020 flow diagram. (DOCX)

S3 Appendix C PRISMA 2020 checklist. (DOCX)

S4 Appendix D AMSTAR 2 checklist. (DOCX)

S5 Appendix E Summary of ROBIS phase 2 and 3 assessments. (DOCX)

S6 Appendix F List of excluded articles with explanation. (DOCX)
