# Supplementary material for: The safety and feasibility of transoral thyroidectomy vestibular approach in the treatment of thyroid disorders: An overview of systematic reviews
Source: PLoS One. 2025 Jul 2;20(7):e0326318. doi: 10.1371/journal.pone.0326318 (PMC12221064; doi:10.1371/journal.pone.0326318)
Supplement: S1 Appendix A — (DOCX) [file pone.0326318.s001.docx]

Appendix A: Search strategy.

|  | **Database** | **Search strategy syntax** |
| --- | --- | --- |
| 1 | PubMed | ((((((transoral thyroidectomy[MeSH Terms]) OR (transoral thyroid surgery[Title/Abstract]))OR (transoral vestibular [Title/Abstract])) OR (transoral vestibule [Title/Abstract])) OR (transoral endoscopes[Title/Abstract])) OR (transoral endoscopic[Title/Abstract]))AND ((((Systematic review[Title/Abstract]) OR (Systematic evaluation[Title/Abstract])) OR (Meta-analysis[Title/Abstract])) OR (meta-analyses[Title/Abstract])) |
| 2 | Cochrane Library | (transoral thyroidectomy or transoral thyroid surgery or transoral vestibular or transoral vestibule or transoral endoscopes or transoral endoscopic):ti,ab,kw AND (Systematic review or Systematic evaluation or Meta-analysis or meta-analyses):ti,ab,kw |
| 3 | EMBASE | ('transoral thyroidectomy':ab,ti OR 'transoral thyroid surgery':ab,ti OR transoral vestibular:ab,ti OR transoral vestibule:ab,ti OR 'transoral endoscopes':ab,ti OR 'transoral endoscopic':ab,ti) AND ('systematic review':ab,ti OR 'systematic evaluation':ab,ti OR 'meta analysis':ab,ti OR 'meta analyses':ab,ti) |
| 4 | Web of Science | (TS=(transoral thyroidectomy OR transoral thyroid surgery OR transoral vestibular OR transoral vestibule OR transoral endoscopes OR transoral endoscopic)) AND (TS=(Systematic review OR Systematic evaluation OR Meta-analysis OR meta-analyses)) |
| 5 | CNKI | (TKA=（经口腔前庭入路+经口腔前庭）+甲状腺切除术) AND (TKA=系统综述 + 系统评价 + 系统评估 + Meta分析) |
| 6 | Wanfang | 题名或关键词:(("经口腔前庭入路" or "经口腔前庭" ) AND"甲状腺切除术") AND 题名或关键词:("系统综述" or "系统评价" or "系统评估" or "Meta分析") |
| 7 | VIP | (M=(经口腔前庭入路 OR 经口腔前庭)AND (M=甲状腺切除术)) AND (M=(系统综述 OR 系统评价 OR 系统评估 OR Meta分析)) |
