# Supplementary material for: The safety and feasibility of transoral thyroidectomy vestibular approach in the treatment of thyroid disorders: An overview of systematic reviews
Source: PLoS One. 2025 Jul 2;20(7):e0326318. doi: 10.1371/journal.pone.0326318 (PMC12221064; doi:10.1371/journal.pone.0326318)
Supplement: S5 Appendix E — (DOCX) [file pone.0326318.s005.docx]

**Appendix E.** Summary of ROBIS Phase 2 and 3 Assessments


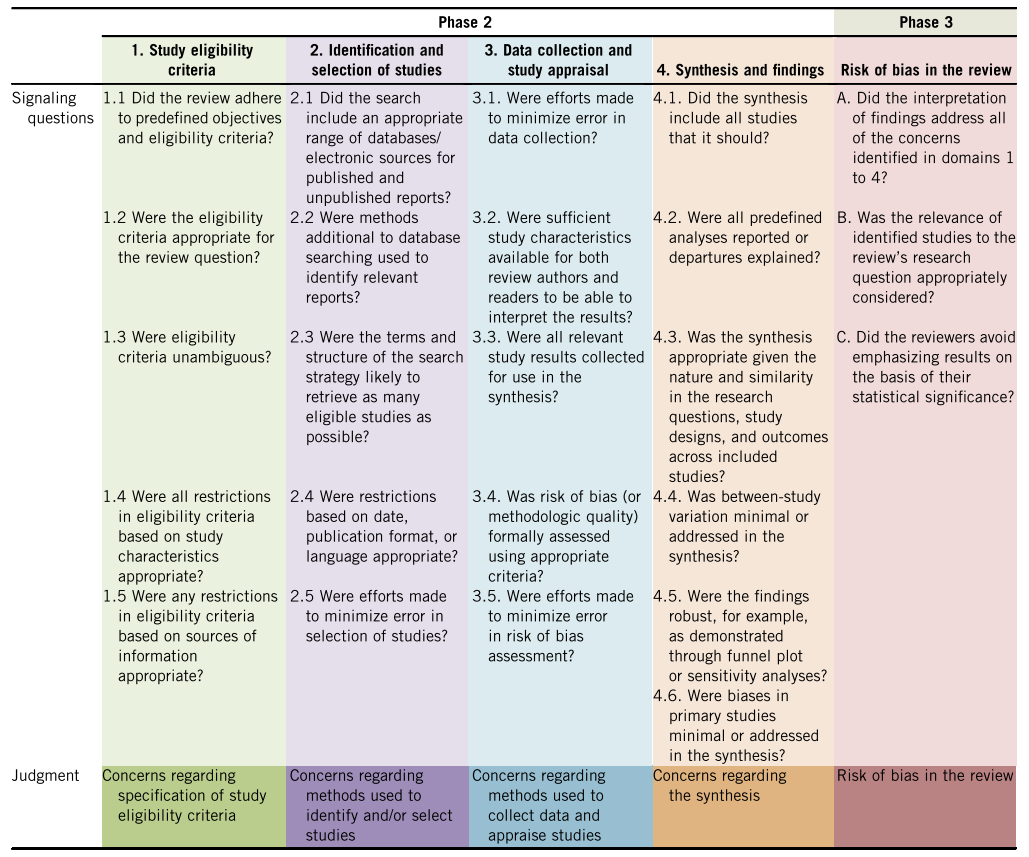


*From:*  Whiting P, Savović J, Higgins JP, et al. ROBIS: A new tool to assess risk of bias in systematic reviews was developed. *J Clin Epidemiol*. 2016;69:225-234. doi:10.1016/j.jclinepi.2015.06.005
