# Supplementary material for: The safety and feasibility of transoral thyroidectomy vestibular approach in the treatment of thyroid disorders: An overview of systematic reviews
Source: PLoS One. 2025 Jul 2;20(7):e0326318. doi: 10.1371/journal.pone.0326318 (PMC12221064; doi:10.1371/journal.pone.0326318)
Supplement: S6 Appendix F — (DOCX) [file pone.0326318.s006.docx]

App**endix F**. List of excluded articles with explanation.

|  | Author (year) | Reference | Reason for exclusion |
| --- | --- | --- | --- |
| 1 | Ahn, S. H., et al(2017) | Guidelines for the Surgical Management of Laryngeal Cancer:Korean Society of Thyroid-Head and Neck Surgery | Other type of related article(guideline) |
| 2 | Akritidou, E., et al(2022) | Complications of Trans-oral Endoscopic Thyroidectomy Vestibular Approach: A Systematic Review | Other type of related article（Complications） |
| 3 | Albalawi, I. A. A., et al. (2023). | The quality of life after trans oral video-assisted thyroidectomy and cervical thyroidectomy: a systematic review and meta-analysis | PICO is inconsistent with the design of this study |
| 4 | Al-lami, A., et al(2022). | Reducing the unknowns: A systematic review & meta-analysis of the effectiveness of trans-oral surgical techniques in identifying head and neck primary cancer in carcinoma unknown primary | Unrelated article |
| 5 | Al-Moraissi, E. A., et al(2018). | Does the surgical approach for treating mandibular condylar fractures affect the rate of seventh cranial nerve injuries? A systematic review and meta-analysis based on a new classification for surgical approaches | Unrelated article |
| 6 | Alonso, E. P., et al(2020). | Early glottic tumours with anterior commissure involvement. Literature review and consensus document. Head and Neck and Skull Base Commission, SEORL-CCC | Other type of related article(review) |
| 7 | Arboleda, L. P. A., et al (2023). | Overview of glottic laryngeal cancer treatment recommendation changes in the NCCN guidelines from 2011 to 2022 | Other type of related article(recommendation) |
| 8 | Baijens, L. W. J., et al(2021). | European white paper: oropharyngeal dysphagia in head and neck cancer. | Unrelated article |
| 9 | Bazarbashi, A. N., et al(2022). | Endoscopic revision of gastrojejunal anastomosis for the treatment of dumping syndrome in patients with Roux-en-Y gastric bypass: a systematic review and meta-analysis | Unrelated article |
| 10 | Beahm, D. D., et al(2009). | Surgical approaches to the submandibular gland: A review of literature | Other type of related article(review) |
| 11 | Belotti, F., et al(2021). | Transclival approaches for intradural pathologies: historical overview and present scenario | Other type of related article(review) |
| 12 | Beumer, L. J., et al(2023). | Success rate of sialendoscopy. A systematic review and meta-analysis | Unrelated article |
| 13 | Blume, M., et al (2019). | Outcome of Surgically Treated Fractures of the Condylar Process by an Endoscopic Assisted Transoral Approach | PICO is inconsistent with the design of this study |
| 14 | Brunaldi, V. O., et al(2018). | Endoscopic Treatment of Weight Regain Following Roux-en-Y Gastric Bypass: a Systematic Review and Meta-analysis | Unrelated article |
| 15 | Camenzuli, C., et al(2018). | Transoral Endoscopic Thyroidectomy: A Systematic Review of the Practice So Far | Other type of related article(review) |
| 16 | Chang, K. J. (2019). | Endoscopic foregut surgery and interventions: The future is now. The state-of-the-art and my personal journey | Unrelated article |
| 17 | Chen, S., et al(2019). | Transoral vestibule approach for thyroid disease: a systematic review | PICO is inconsistent with the design of this study |
| 18 | Chiesa-Estomba, et al(2020). | Imaging checklist for preoperative evaluation of laryngeal tumors to be treated by transoral microsurgery: guidelines from the European Laryngological Society | Other type of related article(guideline) |
| 19 | Chua, T. Y., et al(2021) | GIE Editorial Board top 10 topics: advances in GI endoscopy in 2020 | Unrelated article |
| 20 | Coca, H. A., et al(2023). | Endoscopic endonasal odontoidectomy for basilar invagination in children: Literature review and case report | case report |
| 21 | Colizza, A., et al(2022). | Voice quality after transoral CO<sub>2</sub> laser microsurgery (TOLMS): systematic review of literature | Unrelated article |
| 22 | Crawley, B., et al(2019). | Adverse Events after Rigid and Flexible Endoscopic Repair of Zenker's Diverticula: A Systematic Review and Meta-analysis | Unrelated article |
| 23 | D'Andréa, et al(2022). | Is Transoral Robotic Surgery the Best Surgical Treatment for Lingual Thyroid?: A Case-Report and Literature Review | Other type of related article(review) |
| 24 | Dassé, R., et al(2021) | First-line treatment of exudative vocal fold-lesions by in-office local corticosteroid injection: A literature review | Unrelated article |
| 25 | De Ceulaer, et al(2012). | Robotic surgery in oral and maxillofacial, craniofacial and head and neck surgery: A systematic review of the literature. | Other type of related article(review) |
| 26 | De Moura, et al(2019). | Endoscopic management of weight regain following Roux-en-Y gastric bypass | Unrelated article |
| 27 | de Vries, et al(2021). | Outcomes of Minimally Invasive Thyroid Surgery – A Systematic Review and Meta-Analysis | Unrelated article |
| 28 | Dhindsa, et al(2020). | Efficacy of transoral outlet reduction in Roux-en-Y gastric bypass patients to promote weight loss: a systematic review and meta-analysis | Unrelated article |
| 29 | Dias, F. L., et al(2017). | The role of transoral robotic surgery in the management of oropharyngeal cancer | PICO is inconsistent with the design of this study |
| 30 | Ellsmere, et al(2009). | Endoscopic Interventions for Weight Loss Surgery | Unrelated article |
| 31 | Entezami, et al(2021). | Transoral endoscopic parathyroidectomy vestibular approach: A systematic 32review | Other type of related article(review) |
| 32 | Fang, C. H., et al(2015). | Purely endoscopic endonasal surgery of the craniovertebral junction: A systematic review | Unrelated article |
| 33 | Ferlito, S., et al(2022). | High Definition Three-Dimensional Exoscope (VITOM 3D) in ENT Surgery: A Systematic Review of Current Experience | Unrelated article |
| 34 | Ferrari, D., et al(2021). | Esophageal Lipoma and Liposarcoma: A Systematic Review | Unrelated article |
| 35 | Forastiere, et al(2018). | Use of Larynx-Preservation Strategies in the Treatment of Laryngeal Cancer: American Society of Clinical Oncology Clinical Practice Guideline Update | Other type of related article(guideline) |
| 36 | Franken, et ( al2023). | Efficacy and safety of revisional treatments for weight regain or insufficient weight loss after Roux-en-Y gastric bypass: A systematic review and meta-analysis | Unrelated article |
| 37 | Fuchs, et al(2022). | Results in Antireflux Surgery, an Analysis of Case-Controlled Cohorts versus Multicenter Studies and Meta-Analyses | Unrelated article |
| 38 | Fujii, T., et al(2015). | Endoscopic Endonasal Approaches to the Craniovertebral Junction: A Systematic Review of the Literature | Unrelated article |
| 39 | Gadd, N., et al(2020). | Do Endoscopic Bariatric Procedures Improve Postprocedural Quality of Life and Mental Health? A Systematic Review and Meta-analysis | Unrelated article |
| 40 | Gao, C., et al(2021). | No longer unknown: A systematic review & meta-analysis of the effectiveness of trans-oral surgical techniques in identifying head and neck primary cancer in carcinoma unknown primary | Unrelated article |
| 41 | Garg, R., et al(2022). | Anti-reflux mucosectomy for refractory gastroesophageal reflux disease: a systematic review and meta-analysis | Unrelated article |
| 42 | Gerson, L., et al(2018). | Transoral Incisionless Fundoplication (TIF 2.0): A Meta-Analysis of Three Randomized, Controlled Clinical Trials | Unrelated article |
| 43 | Goh, M. Y. M., et al(2020). | The use of endoluminal techniques in the revision of primary bariatric surgery procedures: a systematic review | Unrelated article |
| 44 | Gong, E. J., et al(2022). | Efficacy of Endoscopic and Surgical Treatments for Gastroesophageal Reflux Disease: A Systematic Review and Network Meta-Analysis | Unrelated article |
| 45 | Gys, B., et al(2019). | Endoscopic Gastric Plication for Morbid Obesity: a Systematic Review and Meta-analysis of Published Data over Time | Unrelated article |
| 46 | Hajjar, A., et al(2023). | Endoscopic plication compared to laparoscopic fundoplication in the treatment of gastroesophageal reflux disease: a systematic review and meta-analysis | Unrelated article |
| 47 | Hartl, D. M., et al(2015). | Management of Retropharyngeal Node Metastases from Thyroid Carcinoma | Unrelated article |
| 48 | Haseeb, M., et al(2023). | Impact of second-generation transoral incisionless fundoplication on atypical GERD symptoms: a systematic review and meta-analysis | Unrelated article |
| 49 | Hillman, L., et al(2017). | Review of antireflux procedures for proton pump inhibitor nonresponsive gastroesophageal reflux disease | Unrelated article |
| 50 | Huang, X. Q., et al(2017). | Efficacy of transoral incisionless fundoplication (TIF) for the treatment of GERD: a systematic review with meta-analysis | Unrelated article |
| 51 | Jaruvongvanich, et al(2020). | Endoscopic full-thickness suturing plus argon plasma mucosal coagulation versus argon plasma mucosal coagulation alone for weight regain after gastric bypass: a systematic review and meta-analysis | Unrelated article |
| 52 | Jeong, et al(2016). | Implants Displaced Into the Maxillary Sinus: A Systematic Review | Unrelated article |
| 53 | Kang, et al(2022). | Safety and Efficacy of Transoral Robotic Thyroidectomy for Thyroid Tumor: A Systematic Review and Meta-Analysis | PICO is inconsistent with the design of this study |
| 54 | Kang, et al(2023). | Surgical completeness and safety of minimally invasive thyroidectomy in patients with thyroid cancer: A network meta-analysis | Unrelated article |
| 55 | Kim, et al(2019). | The Effect of Sphenopalatine Block on the Postoperative Pain of Endoscopic Sinus Surgery: A Meta-analysis | Unrelated article |
| 56 | Lai, et al(2022). | Oncological and Functional Outcomes of Transoral Robotic Surgery and Endoscopic Laryngopharyngeal Surgery for Hypopharyngeal Cancer: A Systematic Review | PICO is inconsistent with the design of this study |
| 57 | Lata, T., et al. (2023) | Current management of gastro-oesophageal reflux disease-treatment costs, safety profile, and effectiveness: a narrative review | Unrelated article |
| 58 | Lechien, J. R., et al(2020). | Surgical, clinical and functional outcomes of transoral robotic surgery for supraglottic laryngeal cancers: A systematic review | Unrelated article |
| 59 | Li, N., et al(2023). | Relationship between dysphagia and surgical treatment for supraglottic laryngeal carcinoma: A meta-analysis | Unrelated article |
| 60 | Martinez-Monedero, R., et al(2020). | Methodological Quality of Systematic Reviews and Meta-analyses Published in High-Impact Otolaryngology Journals | Unrelated article |
| 61 | McCarty, T. R., et al(2018). | Efficacy of transoral incisionless fundoplication for refractory gastroesophageal reflux disease: a systematic review and meta-analysis | Unrelated article |
| 62 | Menderico, G. M., et al(2021). | Complications of transoral endoscopic thyroidectomy vestibular approach (TOETVA) | Other type of related article（Complications） |
| 63 | Mok, A., et al(2020). | A Surgical Mouse Model for Advancing Laryngeal Nerve Regeneration Strategies | Unrelated article |
| 64 | Mukewar, S., et al(2014). | Endoscopic Transoral Reduction (TORe) for Treatment of Weight Regain After Roux-en-Y Gastric Bypass (RYGB): A Meta-Analysis | Unrelated article |
| 65 | Nduma, B. N., et al(2023). | Revision Endoscopic Gastroplasty: An Overview and Review of Literature | Unrelated article |
| 66 | Noor, B., et al(2020). | The effect of endoscopic bariatric therapies on diabetes outcomes: A systematic review | Unrelated article |
| 67 | Oh, M. Y., et al(2023). | Transoral endoscopic thyroidectomy vestibular approach vs. transoral robotic thyroidectomy: systematic review and meta-analysis | PICO is inconsistent with the design of this study |
| 68 | Palmisciano, P., et al(2022). | The Impact of C1 Anterior Arch Preservation on Spine Stability After Odontoidectomy: Systematic Review and Meta-Analysis | Unrelated article |
| 69 | Pangal, D. J., et al(2022). | Robotic and robot-assisted skull base neurosurgery: systematic review of current applications and future directions | Unrelated article |
| 70 | Paspala, A., et al(2020). | Robotic-assisted parathyroidectomy and short-term outcomes: a systematic review of the literature | PICO is inconsistent with the design of this study |
| 71 | Pereira, N. M., et al(2022). | Endoscopic management of intralingual thyroglossal duct cysts: Case series and systematic review | Unrelated article |
| 72 | Prisman, E., et al(2015). | Transoral robotic excision of ectopic lingual thyroid: Case series and literature review | Unrelated article |
| 73 | Purnell, P. R., et al(2022). | Minimally invasive treatment of laryngoceles: a systematic review and pooled analysis | Unrelated article |
| 74 | Rausa, E., et al(2023). | Efficacy of laparoscopic Toupet fundoplication compared to endoscopic and surgical procedures for GERD treatment: a randomized trials network meta-analysis | Unrelated article |
| 75 | Richter, J. E., et al(2018). | Efficacy of Laparoscopic Nissen Fundoplication vs Transoral Incisionless Fundoplication or Proton Pump Inhibitors in Patients With Gastroesophageal Reflux Disease: A Systematic Review and Network Meta-analysis | Unrelated article |
| 76 | Russell, J. O., et al(2019). | Transoral Vestibular Thyroidectomy: Current State of Affairs and Considerations for the Future | Unrelated article |
| 77 | Russo, E., et al(2023). | Different surgical approaches in retropharyngeal lymph nodes dissection in head and neck cancer: A systematic review | PICO is inconsistent with the design of this study |
| 78 | Saito, Y., et al(2022). | A scoping review of approaches used for remote-access parathyroidectomy: A contemporary review of techniques, tools, pros and cons | Other type of related article(review) |
| 79 | Sauer, A. B., et al(2022). | Does surgical management still play a role in the management of ectopic lingual thyroid: Institutional experience and systematic review of the literature | Unrelated article |
| 80 | Shankar, R., et al(2022). | Role of Robotics in Non-oropharyngeal Head and Neck Tumours | Unrelated article |
| 81 | Shriver, M. F., et al(2016). | Transoral and transnasal odontoidectomy complications: A systematic review and meta-analysis | PICO is inconsistent with the design of this study |
| 82 | Singh, R., et al(2022). | Association of meningitis and clival canal defect: case illustration, management, and systematic review of the literature | Unrelated article |
| 83 | Slater, B. J., et al(2023). | Multi-society consensus conference and guideline on the treatment of gastroesophageal reflux disease (GERD) | Unrelated article |
| 84 | Soloperto, D., et al(2022). | Congenital Pyriform Sinus Fistula: Systematic Review and Proposal for Treatment Using a Novel Endoscopic Approach | Unrelated article |
| 85 | Tartaglia, F., et al(2020). | Minimally invasive video-assisted thyroidectomy and transoral video-assisted thyroidectomy: A comparison of two systematic reviews | PICO is inconsistent with the design of this study |
| 86 | Tartaglia, F., et al(2018). | Transoral video assisted thyroidectomy: a systematic review | PICO is inconsistent with the design of this study |
| 87 | Tulli, M., et al(2018). | Diagnosis and Treatment of Laryngeal Schwannoma: A Systematic Review | Unrelated article |
| 88 | Unlu, O., et al(2015). | Endoluminal Bariatric Interventions : Where do we stand ? Where are we going ? | Unrelated article |
| 89 | Vargas, E. J., et al(2018). | Transoral outlet reduction with full thickness endoscopic suturing for weight regain after gastric bypass: a large multicenter international experience and meta-analysis | Unrelated article |
| 90 | Verdonck, J., et al(2015). | Systematic review on treatment of Zenker's diverticulum | Unrelated article |
| 91 | Visocchi, M., et al(2015). | Videoassisted anterior surgical approaches to the craniocervical junction: rationale and clinical results | Unrelated article |
| 92 | Wendling, M. R., et al(2013). | Impact of transoral incisionless fundoplication (TIF) on subjective and objective GERD indices: a systematic review of the published literature | Unrelated article |
| 93 | Wilhelm, T., et al(2016). | Transoral endoscopic thyroidectomy: current state of the art-a systematic literature review and results of a bi-center study | Other type of related article(review) |
| 94 | Witzel, K., et al(2018). | Hybrid techniques and patients' safety in implementing transoral sublingual thyroidectomy | Unrelated article |
| 95 | Wopken, K., et al(2018). | Prognostic factors for tube feeding dependence after curative (chemo-) radiation in head and neck cancer: A systematic review of literature | Unrelated article |
| 96 | Xie, P. W., et al(2021). | Efficacy of different endoscopic treatments in patients with gastroesophageal reflux disease: a systematic review and network meta-analysis. | Unrelated article |
| 97 | Xing, Z. C., et al(2021). | Surgical outcomes of different approaches in robotic assisted thyroidectomy for thyroid cancer: A systematic review and Bayesian network meta-analysis | PICO is inconsistent with the design of this study |
| 98 | Yuan, Y., et al(2013). | Surgical treatment of Zenker's diverticulum | Unrelated article |
| 99 | Zhang, D. Q., et al(2021). | Drawbacks of neural monitoring troubleshooting algorithms in transoral endoscopic thyroidectomy | Unrelated article |
| 100 | An X. (2019). | Meta-Analysis of Prophylactic Central Compartment Lymph Node Dissection on the Prognosis of Patients with Papillary Microcarcinoma of the Thyroid | Other type of related article(review) |
| 101 | Bahettin Karlıp. (2014). | Comprehensive Meta-Analysis of the Application Effect of Ultrasonic Scalpel in Thyroidectomy | Unrelated article |
| 102 | Zeng XQ. (2014). | "Meta-Analysis of Total Thyroidectomy versus Conventional Surgery for the Treatment Efficacy of Differentiated Thyroid Cancer | PICO is inconsistent with the design of this study |
| 103 | Chen JQ. (2014). | Meta-Analysis of the Treatment Efficacy of Miccoli's Modified and Open Surgery for Thyroid Diseases | PICO is inconsistent with the design of this study |
| 104 | Chen P. (2020). | A meta-analysis of the clinical efficacy and safety of prophylactic central neck lymph node dissection in cN0 papillary thyroid carcinoma | Unrelated article |
| 105 | Chen WB. (2019). | Meta-analysis of the Value of Intraoperative Nerve Monitoring for Recurrent Thyroid Surgery and Recurrent Laryngeal Nerve Protection | Unrelated article |
| 106 | Chen X. (2013). | Clinical Study of Thyroid Follicular Carcinoma and the Early Diagnostic Significance of Galectin-3 | diagnostic study |
| 107 | Dong YH. (2013). | Analysis of Surgical Treatment in 62 Cases of Thyroid Papillary Carcinoma | Unrelated article |
| 108 | Fan CL. (2018). | Meta-analysis of High-Risk Factors for Post-Thyroidectomy Local Hematoma Formation | PICO is inconsistent with the design of this study |
| 109 | Fan XD, et al. (2018) | Meta-analysis of Prophylactic Central Neck Lymph Node Dissection in Papillary Thyroid Carcinoma for Local Recurrence and Complications | Unrelated article |
| 110 | Fang XH. (2020). | Network Meta-analysis of Different Treatment Approaches for Hyperthyroidism in Women Planning Pregnancy | Unrelated article |
| 111 | Guo Q, et al. (2020). | Systematic Review of the Application Effect of Fibrin Sealant in Thyroidectomy | Unrelated article |
| 112 | Han H. (2011). | Systematic Review of the Clinical Efficacy of Minimally Invasive Video-Assisted Thyroidectomy Compared to Conventional Open Thyroidectomy | PICO is inconsistent with the design of this study |
| 113 | Li CJ, et al. (2020). | Meta-analysis of the Safety and Short-Term Efficacy of Microwave Ablation vs. Traditional Open Surgery for the Treatment of Thyroid Micro Papillary Carcinoma | PICO is inconsistent with the design of this study |
| 114 | Li C, et al(2014) | Meta-analysis Comparing Recurrence Rates and Complications of Different Surgical Approaches in the Primary Treatment of Differentiated Thyroid Cancer | Unrelated article |
| 115 | Li DW, et al. (2012). | Meta-analysis of Surgical Approach Selection for Differentiated Thyroid Cancer | Unrelated article |
| 116 | Li PX, et al. (2023). | Clinical Characteristics and Predictive Strategies for Non-Recurrent Laryngeal Nerves in Thyroid Surgery | Unrelated article |
| 117 | Li SJ. (2021) | Meta-analysis of Robot-Assisted Thyroidectomy via Bilateral Axillary and Areolar Approach versus Traditional Open Surgery | PICO is inconsistent with the design of this study |
| 118 | Li WH, et al. (2012) | Systematic Review of Endoscopic Thyroidectomy Compared to Conventional Thyroidectomy | PICO is inconsistent with the design of this study |
| 119 | Liu C, et al. (2020) | Meta-analysis of Thermal Ablation and Open Surgery for the Treatment of Thyroid Micro Papillary Carcinoma | PICO is inconsistent with the design of this study |
| 120 | Liu JH, et al. (2016) | Meta-analysis of the Effectiveness and Safety of Robot-assisted Thyroidectomy and Laparoscopic Thyroidectomy for the Treatment of cN0-stage Differentiated Thyroid Cancer | PICO is inconsistent with the design of this study |
| 121 | Liu JH, et al. (2016) | Meta-analysis of the Effectiveness and Safety Comparison Between Minimally Invasive Endoscopic-assisted Thyroidectomy and Traditional Open Thyroidectomy for Treating Lymph Node Negative Thyroid Cancer | PICO is inconsistent with the design of this study |
| 122 | Ma C, et al. (2016) | Meta-analysis of the Short-term Clinical Outcomes of Transcervical Endoscopic Thyroid Surgery and Traditional Open Surgery in the Treatment of Benign Thyroid Tumors | PICO is inconsistent with the design of this study |
| 123 | Ma ZF, et al. (2014) | Meta-analysis of the Impact of Thyroid Surgery on Postoperative Recurrent Laryngeal Nerve Injury in Exposed and Non-exposed Recurrent Laryngeal Nerves | Unrelated article |
| 124 | Meng XM. (2015) | Meta-analysis of the Surgical Treatment of Primary Lesions in Thyroid Papillary Carcinoma | Unrelated article |
| 125 | Ou YJ, et al. (2017) | Meta-analysis of the Comparison of the Effects of Robot-assisted and Traditional Open Thyroidectomy | PICO is inconsistent with the design of this study |
| 126 | Ren BB. (2015) | Comparison of the Therapeutic Efficacy of Laparoscopic-assisted and Fully Laparoscopic Thyroidectomy in the Treatment of Benign Thyroid Nodular Diseases | PICO is inconsistent with the design of this study |
| 127 | Shi Y, et al. (2016) | Meta-analysis of the Safety and Efficacy of Laparoscopic-assisted Thyroidectomy | Unrelated article |
| 128 | Tang JC, et al. (2021) | Meta-analysis of Complications in Total Endoscopic Thyroidectomy for Papillary Thyroid Carcinoma | Unrelated article |
| 129 | Wang ZH, et al. (2008) | Meta-analysis of the Therapeutic Efficacy of Laparoscopic-assisted Thyroidectomy | Unrelated article |
| 130 | Wang JD, et al. (2015) | Meta-analysis of the Therapeutic Efficacy of Laparoscopic-assisted Thyroidectomy | Unrelated article |
| 131 | Wang YD. (2019) | Meta-analysis of Risk Factors for Contralateral Hidden Lobe Nodular Thyroid Carcinoma in Unilateral Micro Papillary Thyroid Carcinoma | Risk factors |
| 132 | Wang ZQ,. (2016) | Meta-analysis of the Clinical Efficacy Comparison between Endoscopic Thyroid Surgery and Traditional Open Thyroid Surgery | PICO is inconsistent with the design of this study |
| 133 | Xie JX. (2022) | Prediction Factors and Meta-analysis of Thyroid Involvement in Locally Advanced Laryngeal Cancer | Risk factors |
| 134 | Yang ZY, et al. (2019) | Meta-analysis of the Efficacy of Ultrasound-guided Percutaneous Microwave Ablation vs. Thyroidectomy for the Treatment of Papillary Microcarcinoma of the Thyroid | PICO is inconsistent with the design of this study |
| 135 | Yerlmak Ahati. (2014). | Systematic Review of Post-Thyroidectomy Parathyroid Hormone Testing for Predicting Hypocalcemia | Unrelated article |
| 136 | You j, et al. (2007). | Evidence-Based Medical Analysis of Routine Drainage in Thyroid and Parathyroid Surgery Postoperatively | Unrelated article |
| 137 | Zhang HT, et al. (2005) | Meta-analysis of the Value of Recurrent Laryngeal Nerve Exposure in Thyroid Surgery | Unrelated article |
| 138 | Zhang H, et al. (2018) | "Meta-analysis of Transareolar Endoscopic Resection vs. Open Surgery for Benign Thyroid Tumors | PICO is inconsistent with the design of this study |
| 139 | Zhang J, et al. (2017) | Meta-analysis of the Efficacy of Minimally Invasive Endoscopic-assisted Thyroidectomy vs. Traditional Approaches in Treating cN_0-stage Papillary Thyroid Carcinoma | PICO is inconsistent with the design of this study |
| 140 | Zhang J, et al. (2013) | Comparative Study of Transareolar Endoscopic Thyroid Surgery and Traditional Surgery | PICO is inconsistent with the design of this study |
| 141 | Zhang TY. (2013) | Safety Systematic Evaluation of Ultrasonic Scalpel and High-Frequency Electric Knife in Thyroidectomy | PICO is inconsistent with the design of this study |
| 142 | Zheng K. (2020) | Meta-analysis of Robot-Assisted Bilateral Axillary Approach Surgery Compared to Open Surgery for Treating Differentiated Thyroid Cancer | PICO is inconsistent with the design of this study |
| 143 | Zhou YC, et al. (2012) | Meta-analysis of the Safety and Efficacy of Cervical Endoscopic Thyroidectomy | Other type of related article(review) |
| 144 | Zhou ZH, et al. (2017) | Meta-analysis of Recurrent Laryngeal Nerve Monitoring in Thyroid Surgery for the Prevention of Nerve Injury | Other type of related article(review) |
| 145 | Zhu SQ. (2021) | Meta-analysis of the Value of Nerve Monitoring Techniques in Protecting the Recurrent Laryngeal Nerve During Thyroid Cancer Surgery | Other type of related article(review) |
| 146 | Zhuang DY, et al. (2021) | Application of the Da Vinci Robot in Pediatric and Adolescent Thyroid Cancer | Unrelated article |
| 147 | Zhuang JZ, et al. (2023) | The translation of the provided text is: "Efficacy and Safety Meta-Analysis of Transoral Vestibular Approach vs. Transareolar Approach in the Endoscopic Treatment of Papillary Thyroid Carcinoma | Lack of Postoperative Primary Outcome Measure |
