# Supplementary material for: The safety and feasibility of transoral thyroidectomy vestibular approach in the treatment of thyroid disorders: An overview of systematic reviews
Source: PLoS One. 2025 Jul 2;20(7):e0326318. doi: 10.1371/journal.pone.0326318 (PMC12221064; doi:10.1371/journal.pone.0326318)
Supplement: Attachment — Submitted filename: Response to Reviewers.docx [file pone.0326318.s009.docx]

We would like to thank the reviewer forcareful and thorough reading of this manuscript and for the thoughtful commentsand constructive suggestions, which help to improve the quality of this manuscript. The manuscript has been rechecked and the necessary changes have been made in accordance with the reviewers’ suggestions. Our response follows:

|  | **Suggestion** | **Response** | **Position** |
| --- | --- | --- | --- |
| 1 | English editing is needed. There are some grammar errors throughout the manuscript. | Thanks for your suggestion. We feel sorry for our poor writings. However, we have proofread the revised manuscript and sent it to an English editing service company. These changes will not influence the content and framework of the paper. And here we did not list the changes but marked in red in revised paper | Changes to the manuscript are shown in red. |
| 2 | The part of literature screening and data extraction, version of the software should be provided. | We feel sorry that we did not provide enough information about the version of the software. We have supplemented the version information of the Endnote software used. | The changes are marked in red on line 123 of the manuscript. |
| 3 | In eligibility criteria, please clarify whether systematic reviews including both RCTs and nonrandomized studies were included. | In eligibility criteria, we have added a description of all the eligible control types: "SRs/MAs containing both randomized controlled trials (RCTs) and non-randomized studies were included". | The changes are marked in red on lines 96-97 of the manuscript. |
| 4 | It is not appropriate to use GRADE to assess the whole quality of SR. GRADE is appropriate to evaluate a body of evidence that usually included in meta-analyses. Please consider rewording evidence quality section. | Thanks for your correction. After searching and studying the literature, we believe that your suggestion is correct, so we have removed the GRADE evaluation content from the manuscript and table. | The changes are marked in red on lines 28-35, 208-225, and table 5 of the manuscript. |
| 5 | The discussion section can expand the advantages of TOTVA in treating thyroid-related diseases. | Thank you for your reminding. We have added the content of the advantages of TOTVA in treating thyroid-related diseases. We have revised the sentences"TOTVA is through a central-median approach with a top-down surgical view, which can preferably expose the thyroid gland, parathyroid glands, and other tissue without obstructing the sternum and clavicle...we believe that TOTVA is a valuable alternative surgical option to COT/NTET with good optional results and excellent cosmetic effects". | The changes are marked in red on lines 286-296 of the manuscript. |
| 6 | It seems that we have not received the attachment you uploaded. Please try uploading again or provide more details. | Thank you for your reminding. I apologize for the inconvenience. There seems to be a problem with the attachment upload. We have re-uploaded the attachment as requested. | In the file “Appendix” |
| 7 | Quality of Included Studies: The quality of studies included in the manuscript may not be sufficiently high to support the conclusions drawn, particularly concerning issues of safety. It's essential to critically evaluate the methodological rigor of the studies and consider whether they provide robust evidence for the conclusions reached. | Thank you for your constructive comments. We recognize that it is crucial to ensure the methodological rigor of the included studies for drawing reliable conclusions, especially regarding issues concerning safety. Therefore, we have expanded the discussion section to include a detailed analysis of limitations related to the methodological quality of the studies. Additionally, we have made specific recommendations for future research. | The changes are marked in red on lines 274-280, 312-315 of the manuscript. |
| 8 | Clarity in Participant Classification: The methods section lacks clarity regarding the classification of participants, particularly concerning thyroid disorders. It's imperative to clearly define and classify the study population based on specific thyroid conditions to facilitate a more nuanced discussion. This classification could include subgroups such as hypothyroidism, hyperthyroidism, thyroid nodules, thyroid cancer, etc. By stratifying the analysis according to these subgroups, it would be possible to explore potential differences in outcomes or treatment responses among different thyroid conditions. | Thank you for your valuable comments. We greatly appreciate your concerns regarding the clarity in participant classification for thyroid diseases. We fully understand the importance of categorizing and refining the classification of thyroid disease types. However, due to the fact that the original studies included in the SRs/MAs we reviewed did not differentiate between malignant and benign thyroid disorders, we can not provide detailed participant classification in the methods section. Additionally, this limitation prevents researchers from performing subgroup analyses on the outcomes of TOTVA and COT/NTET treatments for different thyroid conditions. We have acknowledged and discussed this limitation in the discussion section, exploring its potential impact on the results. Furthermore, future research should place emphasis on the detailed participant classification to reduce publication bias. Ultimately, we hope these explanations and revisions address your concerns. | The changes are marked in red on line 280-284, 300-306 of the manuscript. |
