# Supplementary material for: The safety and feasibility of transoral thyroidectomy vestibular approach in the treatment of thyroid disorders: An overview of systematic reviews
Source: PLoS One. 2025 Jul 2;20(7):e0326318. doi: 10.1371/journal.pone.0326318 (PMC12221064; doi:10.1371/journal.pone.0326318)
Supplement: Attachment — Submitted filename: Manuscript-REVIEW.docx [file pone.0326318.s008.docx]

Dear Editor,

I have read the article “**The Safety and Feasibility of Transoral Thyroidectomy Vestibular Approach in the Treatment of Thyroid Disorders: An Overview of Systematic Reviews**” with interest and found it an interesting and timely demanding issue. However, in this manuscript, the following observations/suggestions require to be clarified before accepting for publication:

1. In general, this is a well-written manuscript evaluating the feasibility and safety of trans-oral thyroid surgery.
2. The title of the manuscript is appropriate and suitable.
3. The abstract includes the background, method, results, and conclusion and is an appropriate summary of the manuscript.
4. The Keywords had a missing transoral thyroidectomy
5. Aims & Objectives are missing in the main manuscript
6. The introduction is appropriately written in the manuscript; In line 54, the author has included the transoral approach. However, he mentioned in lines 55 & 56 the problem of scaring at the incision site.
7. The Efficacy evaluation result section and Table 5 showed six studies compared the trans-oral endoscopic to conventional open, and five studies compared the transoral versus non-trans-oral thyroid surgery, out of which two studies compared robotic thyroid surgery with robotic and endoscopic.
8. based on the above heterogeneous comparison, the author concluded the study, which seems to be inappropriate.
9. References included recent studies and appropriate ones.

Dr. Gyan Chand

Professor, Endocrine Surgery

SGPGIMS, Lucknow.
