# Supplementary material for: The safety and feasibility of transoral thyroidectomy vestibular approach in the treatment of thyroid disorders: An overview of systematic reviews
Source: PLoS One. 2025 Jul 2;20(7):e0326318. doi: 10.1371/journal.pone.0326318 (PMC12221064; doi:10.1371/journal.pone.0326318)
Supplement: Attachment — Submitted filename: Response_to_Reviewers_auresp_2.docx [file pone.0326318.s010.docx]

We would like to thank the reviewer forcareful and thorough reading of this manuscript and for the thoughtful commentsand constructive suggestions, which help to improve the quality of this manuscript. The manuscript has been rechecked and the necessary changes have been made in accordance with the reviewers’ suggestions. Our response follows:

|  | **Suggestion** | **Response** | **Position** |
| --- | --- | --- | --- |
| 1 | In general, this is a well-written manuscript evaluating the feasibility and safety of trans-oral thyroid surgery. | Thank you very much for your positive evaluation of our manuscript. We appreciate your recognition of our work. |  |
| 2 | The title of the manuscript is appropriate and suitable. | We sincerely thank the reviewer for affirming the appropriateness and suitability of the title. |  |
| 3 | The abstract includes the background, method, results, and conclusion and is an appropriate summary of the manuscript. | Thank you for your positive feedback on the structure and content of the abstract. We are glad that it effectively summarizes the manuscript. |  |
| 4 | The Keywords had a missing transoral thyroidectomy. | Thank you for pointing this out. We apologize for not providing sufficient keyword information. We have added "transoral thyroidectomy" to the Keywords section to improve indexing and searchability. | The changes are marked in red on lines 42 of the manuscript. |
| 5 | Aims & Objectives are missing in the main manuscript. | Thank you for your reminder. We have added a paragraph in the Introduction section to clearly state the aims and objectives of our study, thereby clarifying its purpose and focus. We have revised the sentences"This review comprehensively analyzed relevant SRs/MAs to critically assess the safety and feasibility of TOTVA compared with COT and NTET. The objectives were to (1) summarize the efficacy and safety outcomes reported in SRs/MAs comparing TOTVA with COT and NTET, (2) evaluate the methodological quality of the included SRs/MAs, and (3) identify inconsistencies or uncertainties in the evidence to provide a reference for future research and clinical practice." | The changes are marked in red on lines 86-92 of the manuscript. |
| 6 | The introduction is appropriately written in the manuscript; In line 54, the author has included the transoral approach. However, he mentioned in lines 55 & 56 the problem of scaring at the incision site. | Thank you for your careful reading. In the revised manuscript, we have clarified that the issue of visible scarring mainly refers to non-transoral approaches (e.g., areolar, axillary, and retroauricular), not the transoral approach, to avoid potential misunderstanding. We have revised the sentences"Although these techniques, except for the transoral approach, are less invasive than COT, most still require visible external incisions that may result in noticeable scarring[14]. In addition, such approaches may necessitate extensive flap dissection [15], limit access to central neck lymph node dissection [16], or pose challenges in preserving the parathyroid glands during surgery [17].” | The changes are marked in red on lines 51-53 and 56-60 of the manuscript. |
| 7 | The Efficacy evaluation result section and Table 5 showed six studies compared the trans-oral endoscopic to conventional open, and five studies compared the transoral versus non-trans-oral thyroid surgery, out of which two studies compared robotic thyroid surgery with robotic and endoscopic. | Thank you for highlighting this point. Following your suggestion, we have reorganized Table 5 into two separate tables (Table 5A and Table 5B) to clearly distinguish between comparisons:  Table 5A: TOTVA vs. COT  Table 5B: TOTVA vs. NTET (including robotic and non-robotic surgeries). | The changes have been highlighted in red in Table 5A, Table 5B of the manuscript. |
| 8 | Based on the above heterogeneous comparison, the author concluded the study, which seems to be inappropriate. | Thank you for your valuable comment. In the revised version, we have reworded the conclusion to avoid overgeneralization. We now emphasize the tentative nature of the findings and advise cautious interpretation due to heterogeneity, low methodological quality, and risk of bias among included studies. | The changes are marked in red on line 247-250, 310-313 of the manuscript. |
| 9 | References included recent studies and appropriate ones. | We thank the reviewer for the positive feedback regarding the recency and appropriateness of the cited references. |  |
| 10 | In line 21 of the study, it says that the majority of the literature was from 2020 to 2023, but in line 120, the methods section says that the 2023 was from inception to Dec. 10, please explain the reason for this difference. | We appreciate the reviewer’s careful observation. In the Methods section, we clarified that the database search was conducted from inception to December 10, 2023. However, all included SRs/MAs meeting the eligibility criteria were published between 2020 and 2023. We have revised the text at line 21 to explicitly state this to avoid confusion. | The changes are marked in red on line 21-23 of the manuscript. |
| 11 | The reference cited in the preface is incorrectly formatted. Please correct it. | Thank you for your reminder. We have carefully reviewed and corrected the reference formatting according to the journal's guidelines. |  |
| 12 | The English expression of the article is not accurate enough. Please find someone who speaks English to polish it. | Thank you for your valuable comment. We have thoroughly revised the manuscript for language and grammatical accuracy. Two native English speakers have polished the text to improve its clarity and fluency, and the polishing certificate has been attached. | The revised sections are highlighted in blue throughout the manuscript. |
