# Supplementary material for: The safety and feasibility of transoral thyroidectomy vestibular approach in the treatment of thyroid disorders: An overview of systematic reviews
Source: PLoS One. 2025 Jul 2;20(7):e0326318. doi: 10.1371/journal.pone.0326318 (PMC12221064; doi:10.1371/journal.pone.0326318)
Supplement: Peer review history

# Peer review: Decision Letter 0

**DOI:** 10.1371/journal.pone.0326318.r001

**Authors:** Sethu Subha

14 May 2024

Dear Dr. Wang,

Thank you for submitting your manuscript to PLOS ONE. After careful consideration, we feel that it has merit but does not fully meet PLOS ONE’s publication criteria as it currently stands. Therefore, we invite you to submit a revised version of the manuscript that addresses the points raised during the review process.

Please submit your revised manuscript by Jun 28 2024 11:59PM. If you will need more time than this to complete your revisions, please reply to this message or contact the journal office at plosone@plos.org . When you're ready to submit your revision, log on to https://www.editorialmanager.com/pone/ and select the 'Submissions Needing Revision' folder to locate your manuscript file.

- A rebuttal letter that responds to each point raised by the academic editor and reviewer(s). You should upload this letter as a separate file labeled 'Response to Reviewers'.
- A marked-up copy of your manuscript that highlights changes made to the original version. You should upload this as a separate file labeled 'Revised Manuscript with Track Changes'.
- An unmarked version of your revised paper without tracked changes. You should upload this as a separate file labeled 'Manuscript'.

If applicable, we recommend that you deposit your laboratory protocols in protocols.io to enhance the reproducibility of your results. Protocols.io assigns your protocol its own identifier (DOI) so that it can be cited independently in the future. For instructions see: https://journals.plos.org/plosone/s/submission-guidelines#loc-laboratory-protocols . Additionally, PLOS ONE offers an option for publishing peer-reviewed Lab Protocol articles, which describe protocols hosted on protocols.io. Read more information on sharing protocols at https://plos.org/protocols?utm_medium=editorial-email&utm_source=authorletters&utm_campaign=protocols .

We look forward to receiving your revised manuscript.

Kind regards,

Sethu Thakachy Subha, M.S

Academic Editor

PLOS ONE

Journal Requirements:

When submitting your revision, we need you to address these additional requirements.

1. Please ensure that your manuscript meets PLOS ONE's style requirements, including those for file naming. The PLOS ONE style templates can be found at 

https://journals.plos.org/plosone/s/file?id=wjVg/PLOSOne_formatting_sample_main_body.pdf and 

https://journals.plos.org/plosone/s/file?id=ba62/PLOSOne_formatting_sample_title_authors_affiliations.pdf

2. We note that the grant information you provided in the ‘Funding Information’ and ‘Financial Disclosure’ sections do not match. 

When you resubmit, please ensure that you provide the correct grant numbers for the awards you received for your study in the ‘Funding Information’ section.

3. Thank you for stating the following financial disclosure: 

"This study was funded by the National key research and development program of China[grant number [2017YFC1703305], and the Science & Technology Development Fund of Tianjin Education Commission for Higher & Education (2022KJ143)"

Please state what role the funders took in the study.  If the funders had no role, please state: "The funders had no role in study design, data collection and analysis, decision to publish, or preparation of the manuscript." 

If this statement is not correct you must amend it as needed. 

Please include this amended Role of Funder statement in your cover letter; we will change the online submission form on your behalf.

4. PLOS requires an ORCID iD for the corresponding author in Editorial Manager on papers submitted after December 6th, 2016. Please ensure that you have an ORCID iD and that it is validated in Editorial Manager. To do this, go to ‘Update my Information’ (in the upper left-hand corner of the main menu), and click on the Fetch/Validate link next to the ORCID field. This will take you to the ORCID site and allow you to create a new iD or authenticate a pre-existing iD in Editorial Manager. Please see the following video for instructions on linking an ORCID iD to your Editorial Manager account: https://www.youtube.com/watch?v=_xcclfuvtxQ

5. Please only include your tables as part of your main manuscript and remove the individual files. Please note that supplementary tables (should remain/ be uploaded) as separate "supporting information" files"

6. Please review your reference list to ensure that it is complete and correct. If you have cited papers that have been retracted, please include the rationale for doing so in the manuscript text, or remove these references and replace them with relevant current references. Any changes to the reference list should be mentioned in the rebuttal letter that accompanies your revised manuscript. If you need to cite a retracted article, indicate the article’s retracted status in the References list and also include a citation and full reference for the retraction notice.

[Note: HTML markup is below. Please do not edit.]

Reviewers' comments:

Reviewer's Responses to Questions

**Comments to the Author**

1. Is the manuscript technically sound, and do the data support the conclusions?

Reviewer #1: Yes

Reviewer #2: Partly

**********

2. Has the statistical analysis been performed appropriately and rigorously?

Reviewer #1: Yes

Reviewer #2: Yes

**********

3. Have the authors made all data underlying the findings in their manuscript fully available?

The PLOS Data policy

Reviewer #1: Yes

Reviewer #2: Yes

**********

4. Is the manuscript presented in an intelligible fashion and written in standard English?

Reviewer #1: Yes

Reviewer #2: Yes

**********

Reviewer #1: This study provides a valuable overview of the SRs/MAs regarding the outcomes of the TOTVA as compared to COT/ NTET. The authors used a comprehensive methodology to evaluate seven databases for relevant systematic reviews and meta-analyses published between 2020 and 2023, including PRISMA, AMSTAR-2, ROBIS, and GRADE tools. This comprehensive methodology ensures a complete assessment of reporting quality, methodological integrity, bias risk, and overall evidence quality. This is a significant result because it shows that current studies support TOTVA, while demonstrating possible benefits such as improved cosmetic outcomes and fewer postoperative problems. However, several issues must be resolved before consideration for publication.

1. English editing is required. There are several grammar problems in the manuscript.

2. The part of literature screening and data extraction, version of the software should be provided.

3. In eligibility criteria, please clarify whether systematic reviews including both RCTs and nonrandomized studies were included.

4. It is not appropriate to use GRADE to assess the whole quality of SR. GRADE is appropriate

to evaluate a body of evidence that usually included in meta-analyses. Please consider rewording evidence quality section.

5. The discussion section can expand the advantages of TOTVA in treating thyroid-related diseases.

6. It seems that we have not received the attachment you uploaded. Please try uploading again or provide more details.

Reviewer #2: The Transoral Vestibular Approach Thyroidectomy (TOTVA) is a novel surgical technique for thyroidectomy, whereby access to the thyroid region is achieved directly through the oral vestibule, circumventing the need for conventional neck incisions. A systematic review of this surgical approach suggests its potential as a comprehensive option for managing thyroid disorders. The method's advantages lie in reducing postoperative scarring and discomfort in the neck region, thereby facilitating postoperative recovery. This systematic review represents the inaugural comprehensive assessment of the TOTVA for the treatment of thyroid disorders.

The current manuscript still exhibits several key issues that need to be addressed:

1.Quality of Included Studies: The quality of studies included in the manuscript may not be sufficiently high to support the conclusions drawn, particularly concerning issues of safety. It's essential to critically evaluate the methodological rigor of the studies and consider whether they provide robust evidence for the conclusions reached.

2.Clarity in Participant Classification: The methods section lacks clarity regarding the classification of participants, particularly concerning thyroid disorders. It's imperative to clearly define and classify the study population based on specific thyroid conditions to facilitate a more nuanced discussion. This classification could include subgroups such as hypothyroidism, hyperthyroidism, thyroid nodules, thyroid cancer, etc. By stratifying the analysis according to these subgroups, it would be possible to explore potential differences in outcomes or treatment responses among different thyroid conditions.

Addressing these issues will enhance the overall quality and clarity of the manuscript, providing a more robust foundation for the conclusions drawn.

**********

what does this mean? ). If published, this will include your full peer review and any attached files.

If you choose “no”, your identity will remain anonymous but your review may still be made public.

**Do you want your identity to be public for this peer review?** For information about this choice, including consent withdrawal, please see our Privacy Policy

Reviewer #1: No

Reviewer #2: No

**********

[NOTE: If reviewer comments were submitted as an attachment file, they will be attached to this email and accessible via the submission site. Please log into your account, locate the manuscript record, and check for the action link "View Attachments". If this link does not appear, there are no attachment files.]

While revising your submission, please upload your figure files to the Preflight Analysis and Conversion Engine (PACE) digital diagnostic tool, https://pacev2.apexcovantage.com/ . PACE helps ensure that figures meet PLOS requirements. To use PACE, you must first register as a user. Registration is free. Then, login and navigate to the UPLOAD tab, where you will find detailed instructions on how to use the tool. If you encounter any issues or have any questions when using PACE, please email PLOS at figures@plos.org

# Author comment: Author response to Decision Letter 1

**DOI:** 10.1371/journal.pone.0326318.r002

17 Jun 2024

Respond to reviewer #1:

1. Thanks for your suggestion. We feel sorry for our poor writings. However, we have proofread the revised manuscript and sent it to an English editing service company. These changes will not influence the content and framework of the paper. And here we did not list the changes but marked in red in revised paper

2. We feel sorry that we did not provide enough information about the version of the software. We have supplemented the version information of the Endnote software used.

3. In eligibility criteria, we have added a description of all the eligible control types: "SRs/MAs containing both randomized controlled trials (RCTs) and non-randomized studies were included".

4. Thanks for your correction. After searching and studying the literature, we believe that your suggestion is correct, so we have removed the GRADE evaluation content from the manuscript and table.

5. Thank you for your reminding. We have added the content of the advantages of TOTVA in treating thyroid-related diseases. We have revised the sentences"TOTVA is through a central-median approach with a top-down surgical view, which can preferably expose the thyroid gland, parathyroid glands, and other tissue without obstructing the sternum and clavicle...we believe that TOTVA is a valuable alternative surgical option to COT/NTET with good optional results and excellent cosmetic effects".

6. Thank you for your reminding. I apologize for the inconvenience. There seems to be a problem with the attachment upload. We have re-uploaded the attachment as requested.

Respond to reviewer #2:

1. Thank you for your constructive comments. We recognize that it is crucial to ensure the methodological rigor of the included studies for drawing reliable conclusions, especially regarding issues concerning safety. Therefore, we have expanded the discussion section to include a detailed analysis of limitations related to the methodological quality of the studies. Additionally, we have made specific recommendations for future research.

2. Thank you for your valuable comments. We greatly appreciate your concerns regarding the clarity in participant classification for thyroid diseases. We fully understand the importance of categorizing and refining the classification of thyroid disease types. However, due to the fact that the original studies included in the SRs/MAs we reviewed did not differentiate between malignant and benign thyroid disorders, we can not provide detailed participant classification in the methods section. Additionally, this limitation prevents researchers from performing subgroup analyses on the outcomes of TOTVA and COT/NTET treatments for different thyroid conditions. We have acknowledged and discussed this limitation in the discussion section, exploring its potential impact on the results. Furthermore, future research should place emphasis on the detailed participant classification to reduce publication bias. Ultimately, we hope these explanations and revisions address your concerns.

Attachment Submitted filename: Response to Reviewers.docx

# Peer review: Decision Letter 1

**DOI:** 10.1371/journal.pone.0326318.r003

**Authors:** Sethu Subha

22 Apr 2025

Dear Dr. Wang,

Thank you for submitting your manuscript to PLOS ONE. After careful consideration, we feel that it has merit but does not fully meet PLOS ONE’s publication criteria as it currently stands. Therefore, we invite you to submit a revised version of the manuscript that addresses the points raised during the review process.

Please submit your revised manuscript by Jun 06 2025 11:59PM. If you will need more time than this to complete your revisions, please reply to this message or contact the journal office at plosone@plos.org . When you're ready to submit your revision, log on to https://www.editorialmanager.com/pone/ and select the 'Submissions Needing Revision' folder to locate your manuscript file.

- A rebuttal letter that responds to each point raised by the academic editor and reviewer(s). You should upload this letter as a separate file labeled 'Response to Reviewers'.
- A marked-up copy of your manuscript that highlights changes made to the original version. You should upload this as a separate file labeled 'Revised Manuscript with Track Changes'.
- An unmarked version of your revised paper without tracked changes. You should upload this as a separate file labeled 'Manuscript'.

If applicable, we recommend that you deposit your laboratory protocols in protocols.io to enhance the reproducibility of your results. Protocols.io assigns your protocol its own identifier (DOI) so that it can be cited independently in the future. For instructions see: https://journals.plos.org/plosone/s/submission-guidelines#loc-laboratory-protocols . Additionally, PLOS ONE offers an option for publishing peer-reviewed Lab Protocol articles, which describe protocols hosted on protocols.io. Read more information on sharing protocols at https://plos.org/protocols?utm_medium=editorial-email&utm_source=authorletters&utm_campaign=protocols .

We look forward to receiving your revised manuscript.

Kind regards,

Sethu Thakachy Subha, M.S

Academic Editor

PLOS ONE

Journal Requirements:

Please review your reference list to ensure that it is complete and correct. If you have cited papers that have been retracted, please include the rationale for doing so in the manuscript text, or remove these references and replace them with relevant current references. Any changes to the reference list should be mentioned in the rebuttal letter that accompanies your revised manuscript. If you need to cite a retracted article, indicate the article’s retracted status in the References list and also include a citation and full reference for the retraction notice.

[Note: HTML markup is below. Please do not edit.]

Reviewers' comments:

Reviewer's Responses to Questions

**Comments to the Author**

Reviewer #3: All comments have been addressed

Reviewer #4: (No Response)

**********

2. Is the manuscript technically sound, and do the data support the conclusions?

Reviewer #3: Yes

Reviewer #4: Yes

**********

3. Has the statistical analysis been performed appropriately and rigorously?

Reviewer #3: Yes

Reviewer #4: Yes

**********

4. Have the authors made all data underlying the findings in their manuscript fully available?

The PLOS Data policy

Reviewer #3: Yes

Reviewer #4: Yes

**********

5. Is the manuscript presented in an intelligible fashion and written in standard English?

Reviewer #3: Yes

Reviewer #4: Yes

**********

Reviewer #3: I have read the article “The Safety and Feasibility of Transoral Thyroidectomy Vestibular Approach in the Treatment of Thyroid Disorders: An Overview of Systematic Reviews” with interest and found it an interesting and timely demanding issue. However, in this manuscript, the following observations/suggestions require to be clarified before accepting for publication:

1. In general, this is a well-written manuscript evaluating the feasibility and safety of trans-oral thyroid surgery.

2. The title of the manuscript is appropriate and suitable.

3. The abstract includes the background, method, results, and conclusion and is an appropriate summary of the manuscript.

4. The Keywords had a missing transoral thyroidectomy

5. Aims & Objectives are missing in the main manuscript

6. The introduction is appropriately written in the manuscript; In line 54, the author has included the transoral approach. However, he mentioned in lines 55 & 56 the problem of scaring at the incision site.

7. The Efficacy evaluation result section and Table 5 showed six studies compared the trans-oral endoscopic to conventional open, and five studies compared the transoral versus non-trans-oral thyroid surgery, out of which two studies compared robotic thyroid surgery with robotic and endoscopic.

8. based on the above heterogeneous comparison, the author concluded the study, which seems to be inappropriate.

9. References included recent studies and appropriate ones.

Reviewer #4: In line 21 of the study, it says that the majority of the literature was from 2020 to 2023, but in line 120, the methods section says that the 2023 was from inception to Dec. 10, please explain the reason for this difference. The reference cited in the preface is incorrectly formatted. Please correct it. The English expression of the article is not accurate enough. Please find someone who speaks English to Polish it.

**********

what does this mean? ). If published, this will include your full peer review and any attached files.

If you choose “no”, your identity will remain anonymous but your review may still be made public.

**Do you want your identity to be public for this peer review?** For information about this choice, including consent withdrawal, please see our Privacy Policy

Reviewer #3: **Yes: ** GYAN CHAND

Reviewer #4: **Yes: ** JIXIN CHEN

**********

[NOTE: If reviewer comments were submitted as an attachment file, they will be attached to this email and accessible via the submission site. Please log into your account, locate the manuscript record, and check for the action link "View Attachments". If this link does not appear, there are no attachment files.]

While revising your submission, please upload your figure files to the Preflight Analysis and Conversion Engine (PACE) digital diagnostic tool, https://pacev2.apexcovantage.com/ . PACE helps ensure that figures meet PLOS requirements. To use PACE, you must first register as a user. Registration is free. Then, login and navigate to the UPLOAD tab, where you will find detailed instructions on how to use the tool. If you encounter any issues or have any questions when using PACE, please email PLOS at figures@plos.org

Attachment Submitted filename: Manuscript-REVIEW.docx

# Author comment: Author response to Decision Letter 2

**DOI:** 10.1371/journal.pone.0326318.r004

5 May 2025

Reviewer #3:

1. In general, this is a well-written manuscript evaluating the feasibility and safety of trans-oral thyroid surgery.

Response: Thank you very much for your positive evaluation of our manuscript. We appreciate your recognition of our work.

2. The title of the manuscript is appropriate and suitable.

Response: We sincerely thank the reviewer for affirming the appropriateness and suitability of the title.

3. The abstract includes the background, method, results, and conclusion and is an appropriate summary of the manuscript.

Response: Thank you for your positive feedback on the structure and content of the abstract. We are glad that it effectively summarizes the manuscript.

4. The Keywords had a missing transoral thyroidectomy.

Response: Thank you for pointing this out. We apologize for not providing sufficient keyword information. We have added "transoral thyroidectomy" to the Keywords section to improve indexing and searchability. The changes are marked in red on lines 42 of the manuscript.

5. Aims & Objectives are missing in the main manuscript.

Response: Thank you for your reminder. We have added a paragraph in the Introduction section to clearly state the aims and objectives of our study, thereby clarifying its purpose and focus. We have revised the sentences"This review comprehensively analyzed relevant SRs/MAs to critically assess the safety and feasibility of TOTVA compared with COT and NTET. The objectives were to (1) summarize the efficacy and safety outcomes reported in SRs/MAs comparing TOTVA with COT and NTET, (2) evaluate the methodological quality of the included SRs/MAs, and (3) identify inconsistencies or uncertainties in the evidence to provide a reference for future research and clinical practice." The changes are marked in red on lines 86-92 of the manuscript.

6. The introduction is appropriately written in the manuscript; In line 54, the author has included the transoral approach. However, he mentioned in lines 55 & 56 the problem of scaring at the incision site.

Response: Thank you for your careful reading. In the revised manuscript, we have clarified that the issue of visible scarring mainly refers to non-transoral approaches (e.g., areolar, axillary, and retroauricular), not the transoral approach, to avoid potential misunderstanding. We have revised the sentences"Although these techniques, except for the transoral approach, are less invasive than COT, most still require visible external incisions that may result in noticeable scarring[14]. In addition, such approaches may necessitate extensive flap dissection [15], limit access to central neck lymph node dissection [16], or pose challenges in preserving the parathyroid glands during surgery [17].” The changes are marked in red on lines 51-53 and 56-60 of the manuscript.

7.The Efficacy evaluation result section and Table 5 showed six studies compared the trans-oral endoscopic to conventional open, and five studies compared the transoral versus non-trans-oral thyroid surgery, out of which two studies compared robotic thyroid surgery with robotic and endoscopic.

Response: Thank you for highlighting this point. Following your suggestion, we have reorganized Table 5 into two separate tables (Table 5A and Table 5B) to clearly distinguish between comparisons:

Table 5A: TOTVA vs. COT

Table 5B: TOTVA vs. NTET (including robotic and non-robotic surgeries).

The changes have been highlighted in red in Table 5A, Table 5B of the manuscript.

8. Based on the above heterogeneous comparison, the author concluded the study, which seems to be inappropriate.

Response: Thank you for your valuable comment. In the revised version, we have reworded the conclusion to avoid overgeneralization. We now emphasize the tentative nature of the findings and advise cautious interpretation due to heterogeneity, low methodological quality, and risk of bias among included studies. The changes are marked in red on line 247-250, 310-313 of the manuscript.

9. References included recent studies and appropriate ones.

Response: We thank the reviewer for the positive feedback regarding the recency and appropriateness of the cited references.

Reviewer #4:

1. In line 21 of the study, it says that the majority of the literature was from 2020 to 2023, but in line 120, the methods section says that the 2023 was from inception to Dec. 10, please explain the reason for this difference.

Response: We appreciate the reviewer’s careful observation. In the Methods section, we clarified that the database search was conducted from inception to December 10, 2023. However, all included SRs/MAs meeting the eligibility criteria were published between 2020 and 2023. We have revised the text at line 21 to explicitly state this to avoid confusion. The changes are marked in red on line 21-23 of the manuscript.

2. The reference cited in the preface is incorrectly formatted. Please correct it.

Response: Thank you for your reminder. We have carefully reviewed and corrected the reference formatting according to the journal's guidelines.

3.The English expression of the article is not accurate enough. Please find someone who speaks English to polish it.

Response: Thank you for your valuable comment. We have thoroughly revised the manuscript for language and grammatical accuracy. Two native English speakers have polished the text to improve its clarity and fluency, and the polishing certificate has been attached. The revised sections are highlighted in blue throughout the manuscript.

Attachment Submitted filename: Response_to_Reviewers_auresp_2.docx

# Peer review: Decision Letter 2

**DOI:** 10.1371/journal.pone.0326318.r005

**Authors:** Sethu Subha

28 May 2025

The safety and feasibility of transoral thyroidectomy vestibular approach in the treatment of thyroid disorders: an overview of systematic reviews

PONE-D-24-12039R2

Dear Dr. ,

We’re pleased to inform you that your manuscript has been judged scientifically suitable for publication and will be formally accepted for publication once it meets all outstanding technical requirements.

Within one week, you’ll receive an e-mail detailing the required amendments. When these have been addressed, you’ll receive a formal acceptance letter and your manuscript will be scheduled for publication.

An invoice will be generated when your article is formally accepted. Please note, if your institution has a publishing partnership with PLOS and your article meets the relevant criteria, all or part of your publication costs will be covered. Please make sure your user information is up-to-date by logging into Editorial Manager at Editorial Manager®  and clicking the ‘Update My Information' link at the top of the page. If you have any questions relating to publication charges, please contact our Author Billing department directly at authorbilling@plos.org.

If your institution or institutions have a press office, please notify them about your upcoming paper to help maximize its impact. If they’ll be preparing press materials, please inform our press team as soon as possible -- no later than 48 hours after receiving the formal acceptance. Your manuscript will remain under strict press embargo until 2 pm Eastern Time on the date of publication. For more information, please contact onepress@plos.org.

Kind regards,

Sethu Thakachy Subha, M.S

Academic Editor

PLOS ONE

Additional Editor Comments (optional):

Reviewers' comments:

Reviewer's Responses to Questions

**Comments to the Author**

Reviewer #4: All comments have been addressed

**********

2. Is the manuscript technically sound, and do the data support the conclusions?

Reviewer #4: No

**********

3. Has the statistical analysis been performed appropriately and rigorously?

Reviewer #4: Yes

**********

4. Have the authors made all data underlying the findings in their manuscript fully available?

The PLOS Data policy

Reviewer #4: Yes

**********

5. Is the manuscript presented in an intelligible fashion and written in standard English?

Reviewer #4: Yes

**********

Reviewer #4: I have no further questions about this article. It is a well-written manuscript evaluating the feasibility and safety of

trans-oral thyroid surgery.

**********

what does this mean? ). If published, this will include your full peer review and any attached files.

If you choose “no”, your identity will remain anonymous but your review may still be made public.

**Do you want your identity to be public for this peer review?** For information about this choice, including consent withdrawal, please see our Privacy Policy

Reviewer #4: **Yes: ** Qinxin Zhou

**********

# Editor report: Acceptance letter

**DOI:** 10.1371/journal.pone.0326318.r006

**Authors:** Sethu Subha

PONE-D-24-12039R2

PLOS ONE

Dear Dr. Wang,

I'm pleased to inform you that your manuscript has been deemed suitable for publication in PLOS ONE. Congratulations! Your manuscript is now being handed over to our production team.

At this stage, our production department will prepare your paper for publication. This includes ensuring the following:

* All references, tables, and figures are properly cited

* All relevant supporting information is included in the manuscript submission,

* There are no issues that prevent the paper from being properly typeset

You will receive further instructions from the production team, including instructions on how to review your proof when it is ready. Please keep in mind that we are working through a large volume of accepted articles, so please give us a few days to review your paper and let you know the next and final steps.

Lastly, if your institution or institutions have a press office, please let them know about your upcoming paper now to help maximize its impact. If they'll be preparing press materials, please inform our press team within the next 48 hours. Your manuscript will remain under strict press embargo until 2 pm Eastern Time on the date of publication. For more information, please contact onepress@plos.org.

If we can help with anything else, please email us at customercare@plos.org.

Thank you for submitting your work to PLOS ONE and supporting open access.

Kind regards,

PLOS ONE Editorial Office Staff

on behalf of

Dr. Sethu Thakachy Subha

Academic Editor

PLOS ONE
